# Comparison of carnivore, omnivore, and herbivore mammalian genomes with a new leopard assembly

**DOI:** 10.1186/s13059-016-1071-4

**Published:** 2016-10-11

**Authors:** Soonok Kim, Yun Sung Cho, Hak-Min Kim, Oksung Chung, Hyunho Kim, Sungwoong Jho, Hong Seomun, Jeongho Kim, Woo Young Bang, Changmu Kim, Junghwa An, Chang Hwan Bae, Youngjune Bhak, Sungwon Jeon, Hyejun Yoon, Yumi Kim, JeHoon Jun, HyeJin Lee, Suan Cho, Olga Uphyrkina, Aleksey Kostyria, John Goodrich, Dale Miquelle, Melody Roelke, John Lewis, Andrey Yurchenko, Anton Bankevich, Juok Cho, Semin Lee, Jeremy S. Edwards, Jessica A. Weber, Jo Cook, Sangsoo Kim, Hang Lee, Andrea Manica, Ilbeum Lee, Stephen J. O’Brien, Jong Bhak, Joo-Hong Yeo

**Affiliations:** 1 Biological and Genetic Resources Assessment Division, National Institute of Biological Resources, Incheon, 22689 Republic of Korea; 2 The Genomics Institute, Ulsan National Institute of Science and Technology (UNIST), Ulsan, 44919 Republic of Korea; 3 Department of Biomedical Engineering, School of Life Sciences, Ulsan National Institute of Science and Technology (UNIST), Ulsan, 44919 Republic of Korea; 4 Personal Genomics Institute, Genome Research Foundation, Cheongju, 28160 Republic of Korea; 5 Geromics, Ulsan, 44919 Republic of Korea; 6 Animal Resources Division, National Institute of Biological Resources, Incheon, 22689 Republic of Korea; 7 Cheongju Zoo, Cheongju, 28311 Republic of Korea; 8 Institute of Biology & Soil Science, Far Eastern Branch of Russian Academy of Sciences, Vladivostok, 690022 Russia; 9 Panthera, New York, NY 10018 USA; 10 Wildlife Conservation Society, 2300 Southern Boulevard, Bronx, NY 10460 USA; 11 Department of Ecology, Far Eastern Federal University, Ayaks, Russki Island, Vladivostok, 690950 Russia; 12 Laboratory of Animal Sciences Program, Leídos Biomedical Research Inc., Frederick National Laboratory, Frederick, MD 21702 USA; 13 International Zoo Veterinary Group (UK) IZVG LLP, Station House, Parkwood Street, Keighley, BD21 4NQ UK; 14 Theodosius Dobzhansky Center for Genome Bioinformatics, St. Petersburg State University, St. Petersburg, 199004 Russia; 15 Center for Algorithmic Biotechnology, Institute for Translational Biomedicine, St. Petersburg State University, St. Petersburg, 199034 Russia; 16 Broad Institute of MIT and Harvard, Cambridge, MA 02142 USA; 17 Department of Biomedical Informatics, Harvard Medical School, Boston, MA 02115 USA; 18 Chemistry and Chemical Biology, UNM Comprehensive Cancer Center, University of New Mexico, Albuquerque, NM 87131 USA; 19 Department of Biology, University of New Mexico, Albuquerque, NM 87131 USA; 20 Zoological Society of London, London, NW1 4RY UK; 21 Department of Bioinformatics & Life Science, Soongsil University, Seoul, 06978 Republic of Korea; 22 Conservation Genome Resource Bank for Korean Wildlife, College of Veterinary Medicine, Seoul National University, Seoul, 08826 Republic of Korea; 23 Department of Zoology, University of Cambridge, Downing Street, Cambridge, CB2 3EJ UK; 24 Daejeon O-World, Daejeon, 35073 Republic of Korea; 25 Oceanographic Center 8000 N. Ocean Drive, Nova Southeastern University, Ft Lauderdale, FL 33004 USA

**Keywords:** Carnivorous diet, Evolutionary adaptation, Leopard, Felidae, *De novo* assembly, Comparative genomics

## Abstract

**Background:**

There are three main dietary groups in mammals: carnivores, omnivores, and herbivores. Currently, there is limited comparative genomics insight into the evolution of dietary specializations in mammals. Due to recent advances in sequencing technologies, we were able to perform in-depth whole genome analyses of representatives of these three dietary groups.

**Results:**

We investigated the evolution of carnivory by comparing 18 representative genomes from across Mammalia with carnivorous, omnivorous, and herbivorous dietary specializations, focusing on Felidae (domestic cat, tiger, lion, cheetah, and leopard), Hominidae, and Bovidae genomes. We generated a new high-quality leopard genome assembly, as well as two wild Amur leopard whole genomes. In addition to a clear contraction in gene families for starch and sucrose metabolism, the carnivore genomes showed evidence of shared evolutionary adaptations in genes associated with diet, muscle strength, agility, and other traits responsible for successful hunting and meat consumption. Additionally, an analysis of highly conserved regions at the family level revealed molecular signatures of dietary adaptation in each of Felidae, Hominidae, and Bovidae. However, unlike carnivores, omnivores and herbivores showed fewer shared adaptive signatures, indicating that carnivores are under strong selective pressure related to diet. Finally, felids showed recent reductions in genetic diversity associated with decreased population sizes, which may be due to the inflexible nature of their strict diet, highlighting their vulnerability and critical conservation status.

**Conclusions:**

Our study provides a large-scale family level comparative genomic analysis to address genomic changes associated with dietary specialization. Our genomic analyses also provide useful resources for diet-related genetic and health research.

**Electronic supplementary material:**

The online version of this article (doi:10.1186/s13059-016-1071-4) contains supplementary material, which is available to authorized users.

## Background

Diet is, perhaps, the most serious selection force in all species on Earth. In particular, carnivory is interesting because it has evolved repeatedly in a number of mammalian clades [1, 2]. In the fossil record, specialization in carnivory is often associated with relatively short extinction times, a likely consequence of the small population sizes associated with a diet at the top of the trophic pyramid [1, 2]. Indeed, many carnivore specialists have closely related species that have a much broader diet, such as polar bears, grizzly (omnivore), and panda (herbivore) bears in Ursidae [3, 4] and foxes (omnivore) in Canidae [5], highlighting the frequent evolutionary instability of this lifestyle.

Felidae (cats), together with Mustelidae, are unusual mammalian groups whose members are all obligate carnivores (hypercarnivores) [6]. Specialized diets have resulted in a number of physiological, biochemical, and morphological adaptations. In carnivores, several key diet-related physiological traits have been identified, including differences in digestive enzymes [7], shortened digestive tracts [8], changes in amino acid dietary requirements [9, 10], and alterations to taste bud sensitivities (including a heightened response to amino acids and a loss of response to many mono- and di-saccharides) [11, 12], to name a few. In addition to these characteristics, the morphology of cats is highly adapted to hunting and includes flexible bodies, fast reflexes, and strong muscular limbs. Felids also possess strong night vision and hearing, which are critical for hunting [13, 14]. Felidae is a well-studied group from a genomic perspective: the first cat assembly (*Felis catus*) was released in 2007 and the tiger (*Panthera tigris*) genome assembly was published in 2013, together with lion and snow leopard whole genome data [15, 16]. Subsequently, a high-quality domestic cat reference and a cheetah (*Acinonyx jubatus*) genome assembly have also been added [17–19], making this group an ideal initial target for identifying molecular adaptations to extreme carnivory that can provide insight on human healthcare.

Here, we investigated the genomic adaptations to diets by first expanding genomic coverage of Felidae, producing the highest quality big cat reference genome assembly for leopard (*Panthera pardus*) and whole genome data for leopard cat (*Prionailurus bengalensis*). Leopards are the most widespread species of the big cats (from Africa to the Russian Far East), thriving in a great variety of environments [20]. This leopard assembly provides an additional non-domesticated big cat genome that can be co-analyzed with the most accurate domestic cat genome reference, resulting in reliable genomic scale genetic variation studies across Felidae. These new data allowed us to compare five cat references (domestic cat, tiger, cheetah, lion, and leopard) and two re-sequenced genomes (snow leopard and leopard cat) at a level of coverage comparable to other well studied groups such as hominids and artiodactyls. Taking advantage of this wealth of data, we performed a number of comparative analyses to investigate the molecular adaptations to carnivory.

## Results and discussion

### Leopard genome sequencing and assembly

We built the reference leopard genome from a muscle sample obtained from a female Amur leopard from the Daejeon O-World of Korea (Additional file 1: Supplemental Methods for details of species identification using mitochondrial DNA (mtDNA) gene analysis; Additional file 2: Figure S1). The extracted DNA was sequenced to 310× average depth of coverage using Illumina HiSeq platforms (Additional file 3: Tables S1 and S2). Sequenced reads were filtered and then error-corrected using a *K*-mer analysis. The size of the leopard genome was estimated to be ~2.45 Gb (Additional file 1: Supplemental Methods for details; Additional file 2: Figure S2; Additional file 3: Table S3). The error-corrected reads were assembled using SOAPdenovo2 software [21] into 265,373 contigs (N50 length of 21.0 kb) and 50,400 scaffolds (N50 length of 21.7 Mb), totaling 2.58 Gb in length (Additional file 1: Supplemental Methods for details; Additional file 3: Table S4). Additionally, 393,866 Illumina TruSeq synthetic long reads [22] (TSLRs, 2.0 Gb of total bases; ~0.8×) were obtained from two wild Amur leopard individuals (Additional file 3: Tables S5 and S6) and were used to correct erroneous gap regions. The GC content and distribution of the leopard genome were very similar to those of the tiger and domestic cat genomes (Additional file 2: Figure S3), indicating little sequencing and assembly bias. We successfully predicted 19,043 protein-coding genes for the leopard genome by combining *de novo* and homologous gene prediction methods (Additional file 3: Table S7; see “Methods”). In total, 39.04 % of the leopard genome were annotated as transposable elements (Additional file 1: Supplemental Methods for details; Additional file 3: Table S8), which is very similar in composition to the other felid species [16, 18, 19]. Assembly quality was assessed by aligning the short sequence reads onto the scaffolds (99.7 % mapping rate) and compared with other Felidae species assemblies (cat, tiger, cheetah, and lion) using common assembly metrics (Additional file 3: Tables S9 and S10). The genome assembly and annotation completeness were assessed by the commonly used single-copy ortholog mapping approach [23] (Additional file 3: Table S11). The leopard genome showed the longest continuity and highest accuracy among the big cat (*Panthera* species and cheetah) genome assemblies. Two additional wild Amur leopards from the Russian Far East and a wild Amur leopard cat from Korea were whole genome re-sequenced (Additional file 3: Tables S5 and S12), and were used together with previously reported whole genome data of other felid species [16] for comparative evolutionary analyses.

### Evolutionary analysis of carnivores compared to omnivores and herbivores

To investigate the genomic adaptations to different diets and their associated lifestyles, we performed an extensive orthologous gene comparison among eight carnivorous (leopard, cat, tiger, cheetah, lion, polar bear, killer whale, and Tasmanian devil), five omnivorous (human, mouse, dog, pig, and opossum), and five herbivorous mammalian genomes (giant panda, cow, horse, rabbit, and elephant; Additional file 1: Supplemental Methods for details of species selection criteria; Additional file 3: Table S13). These comparisons revealed numerous genetic signatures consistent with molecular adaptations to a hypercarnivorous lifestyle.

**Fig. 1:**
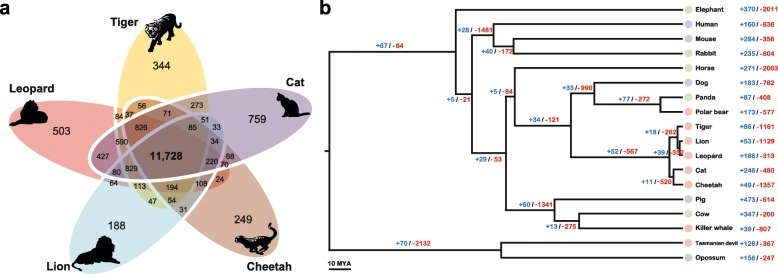
Relationship of Felidae to other mammalian species. **a** Orthologous gene clusters in Felidae species. Orthologous gene clusters were constructed using 18 mammalian genomes. Only Felidae species gene clusters are displayed in this figure. **b** Gene expansion or contraction in mammalian species. Branch numbers indicate the number of gene families that have expanded (*blue*) and contracted (*red*) after the split from the common ancestor. Colors of *circles* represent diet groups (*light red*: carnivore, *light blue*: omnivore, *light green*: herbivore). The *time lines* indicate divergence times among the species

**Fig. 2:**
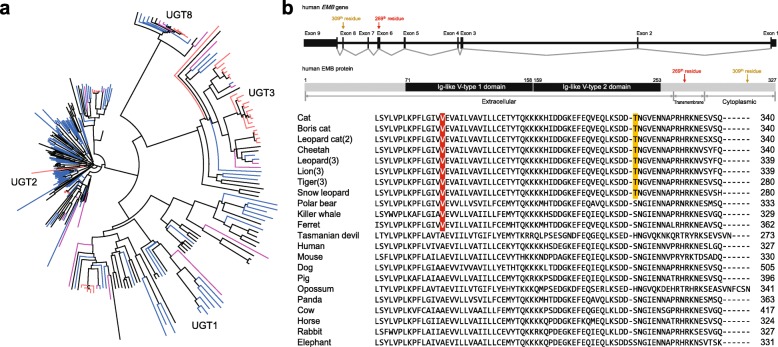
Gene copy evolution and amino acid changes (AACs) in Felidae and carnivores. **a** Contracted (UGT1 and UGT2) and expanded (UGT3) UDP-glucuronosyltransferase families in carnivores. The *red*, *violet*, *blue*, and *black* nodes are UGT family genes in the five cats, non-cat carnivores (polar bear, killer whale, and Tasmanian devil), five herbivores, and five omnivores, respectively. **b** Convergent AAC found in carnivores. Human embigin (*EMB*) gene and predicted protein structures are illustrated in the *upper part*. Amino acids specific to the carnivores (269th residue in human EMB protein, transmembrane region) and felids (309th residue, cytoplasmic region) in EMB protein are shown in *red* and *yellow*, respectively. The *numbers in parentheses* are number of genomes analyzed in this study

Of the 15,589 orthologous gene families found in the leopard assembly, 11,748 were also found in the other four Felidae genomes and 8648 in the complete set of 18 mammalian genomes across all three dietary groups (Fig. 1a and Additional file 2: Figure S4). The leopard genome displayed 188 expanded and 313 contracted gene families compared with the common ancestor of leopard and lion (Fig. 1b and Additional file 2: Figure S5). The common ancestor of Felidae species showed 52 expanded and 567 contracted gene families compared to the common ancestor of carnivorans. In particular, Felidae expanded gene families were enriched in muscle myosin complex (GO:0005859, nine genes, *P* = 1.14 × 10^–13^ by EASE scores [modified Fisher’s exact test] with a 10 % false discovery rate [FDR]) and actin cytoskeleton (GO:0015629, 14 genes, *P* = 4.71 × 10^–9^) functions that are associated with muscle contraction and motor activity (Additional file 3: Tables S14 and S15). Conversely, Felidae clearly showed contracted gene families in starch and sucrose metabolism pathway (*P* = 5.62 × 10^–7^; Additional file 3: Tables S16 and S17). Notably, the common ancestor of the Carnivora order (compared to the common ancestor of carnivorans and horse) and killer whale (compared to the common ancestor of killer whale and cow) also had contracted gene families associated with starch and sucrose metabolism (*P* = 0.0000032 and *P* = 0.00048, respectively; Additional file 3: Tables S18–S25), whereas Tasmanian devil (a well-known scavenger as well as a meat-eating carnivore [24]) did not (compared to the common ancestor of Tasmanian devil and opossum; Additional file 3: Tables S26–S29). UDP-glucuronosyltransferase (UGT) 1 and 2 families playing an important role in detoxification and homeostatic functions were markedly contracted in the carnivores (Fig. 2a and Additional file 3: Table S30). This is in contrast to herbivores that must have acquired detoxification pathways to protect themselves against plant-derived toxicants. It is very likely that the low dietary content of these plant-derived toxicants in carnivores is a major factor in the UGT 1 and 2 contractions in carnivores [25, 26]. However, the UGT3 family, which is involved in the conjugation with *N*-acetylglucosamine and glucose [27], was expanded only in the Felidae genomes. *UGT8A1* that is involved in conjugation of ceramides and bile acids with galactose [28] was conserved (in terms of gene copy number) in all 18 mammals. Additionally and expectedly, amylase gene families (AMY1 and AMY2), which catalyze dietary starch and glycogen, were contracted in the carnivores (Additional file 2: Figure S6; Additional file 3: Table S30), providing a genetic mechanism for the very low levels of salivary amylase observed in cats [29].

It is known that cats lack the ability to synthesize sufficient amounts of vitamin A and arachidonic acid, making them essential [30]. Interestingly, cytochrome P450 (CYP) family genes, which are involved in retinol/linoleic acid/arachidonic acid catabolism, were commonly contracted in all the carnivorous diet-groups (Felidae, Carnivora order, killer whale, and Tasmanian devil; Additional file 3: Tables S18–S29). Retinoic acid converted from retinol is essential for teeth remineralization and bone growth [31, 32] and arachidonic acid promotes the repair and growth of skeletal muscle tissue after physical exercise [33]. We speculate that the contraction of CYP family genes may help carnivores to keep sufficient levels of retinol and arachidonic acid concentration on their body and, therefore, they could have evolved to possess strong muscle, bone, and teeth for successful hunting.

Although carnivores derive their energy and nutrient requirements primarily from animal tissues, they also require regulatory mechanisms to ensure an adequate supply of glucose to tissues, such as the brain [34]. The glucokinase (GCK) enzyme is responsible for regulating the uptake and storage of dietary glucose by acting as a glucose sensor [35]. The mutations in gene for glucokinase regulatory protein (*GCKR*) have effects on glucose and lipid homeostasis; and GCK and glucokinase regulatory protein (GKRP, encoded by *GCKR* gene) have been suggested as a target for diabetes treatment in humans [35]. It was predicted that *GCKR* is pseudogenized by frame-shift mutations in multiple mammalian genomes including cat [36]. We confirmed that *GCKR* is also pseudogenized by frame-shift mutations in all other felids (leopard, tiger, lion, cheetah, snow leopard, and leopard cat; Additional file 2: Figure S7). Interestingly, *GCKR* genes of killer whale and domestic ferret (another obligate carnivore not used in this study) [37] were also pseudogenized by pre-matured and/or frame-shift mutations, whereas polar bear and Tasmanian devil have an intact *GCKR* (Additional file 3: Table S31). It has been suggested that carnivores may not need to remove excess glucose from the circulation, as they consume food containing large amounts of protein and little carbohydrate [36]. Among the non-carnivorous animals, *GCKR* genes of cow and opossum were predicted to be pseudogenized. In the case of cow, it was speculated that ruminant animals use volatile fatty acids generated by fermentation in their foregut as main energy source and they may not need to remove excess glucose actively [36]. Therefore, the evolutionary loss of *GCKR* and the accompanying adaptation of the glucose-sensing pathway to carnivory will help us to better understand the abnormal glucose metabolism that characterizes the diabetic state [34].

To detect genes evolving under selection for a diet specialized in meat, we performed tests for deviations in the *d*_*N*_/*d*_*S*_ ratio (non-synonymous substitutions per non-synonymous site to synonymous substitutions per synonymous site, branch model) and likelihood ratio tests (branch-site model) [38, 39]. A total of 586 genes were identified as positively selected genes (PSGs) in the leopard genome (Additional file 4: Datasheet S1). The leopard PSGs were functionally enriched in GTP binding (GO:0005525, 24 genes, *P* = 0.00013), regulation of cell proliferation (GO:0042127, 39 genes, *P* = 0.00057), and macromolecule catabolic process (GO:0009057, 38 genes, *P* = 0.00096; Additional file 3: Table S32). Additionally, 228 PSGs were shared in the Felidae family (cat, tiger, lion, cheetah, and leopard); we defined shared PSGs as those that are found in two or more species (Additional file 4: Datasheet S2). The shared PSGs of Felidae were enriched in polysaccharide binding (GO:0030247, eight genes, *P* = 0.00071), lipid binding (GO:0008289, 12 genes, *P* = 0.0041), and immune response (GO:0006955, 16 genes, *P* = 0.0052; Additional file 3: Table S33). Since felid species are hypercarnivores [3], selection of the lipid binding associated genes may be associated to their obligatory carnivorous diet and regulation of lipid and cholesterol homeostasis [16, 40]. We further identified shared PSGs in the eight carnivores (PSGs in three or more species), five omnivores (PSGs in two or more species), or five herbivores (PSGs in two or more species). A total of 184, 221, and 136 genes were found as shared PSGs among carnivores, omnivores, and herbivores, respectively (Additional file 4: Datasheets S3–S5). The carnivores’ shared PSGs were significantly enriched in motor axon guidance (GO:0008045, three genes, *P* = 0.0050; Additional file 3: Table S34). *CXCL12* (stromal cell-derived factor 1), which was found as a shared PSG in carnivores, is known to influence the guidance of both migrating neurons and growing axons. *CXCL12*/*CXCR4* signaling has been shown to regulate motor axon projection in the mouse [41, 42]. Two other carnivore-shared PSGs, *DMP1* and *PTN*, are known to play an important role in bone development and repair [43, 44]. In contrast, there was no significant positive selection of the muscle and bone development associated genes in the omnivores and herbivores. Instead, several immune associated functional categories, such as response to cytokine stimulus, cytokine activity, and regulation of leukocyte activation, were enriched in omnivores and herbivores (Additional file 3: Tables S35–S38).

If adaptive evolution affects only a few crucial amino acids over a short time period, none of the methods for measuring selection is likely to succeed in defining positive selection [45]. Therefore, we investigated target species-specific amino acid changes (AACs) using 15 feline (three leopards, three lions, a snow leopard, three tigers, two leopard cats, a cheetah, and two cats; Additional file 3: Table S39) and additional 13 mammalian genomes. A total of 1509 genes in the felids were predicted to have at least one function altering AAC (Additional file 4: Datasheet S6). Unexpectedly but understandably, the Felidae-specific genes with function altering AACs were enriched in response to DNA damage stimulus (GO:0006974, 53 genes, *P* = 7.39 × 10^–7^), DNA repair (GO:0006281, 41 genes, *P* = 0.000011), and cellular response to stress (GO:0033554, 63 genes, *P* = 0.00016; Additional file 2: Figure S8; Additional file 3: Tables S40 and S41). Interestingly, three genes (*MEP1A*, *ACE2*, and *PRCP*), which are involved in the protein digestion and absorption pathway, had function altering AACs specific to Felidae species (Additional file 2: Figures S9–S11). We interpret this result as a dietary adaptation for high meat consumption that is associated with an increased risk of cancer in humans [46], and that the heme-related reactive oxygen species (ROS) in meat cause DNA damage and disrupt normal cell proliferation [47, 48]. We speculate that the functional changes found in DNA damage and repair associated genes help reduce diet-related DNA damage in the felid species. This possible felid’s genetic feature can lead to better understanding of human dietary and health research [34].

We also identified convergent AACs in the carnivores (Felidae, polar bear, killer whale, and Tasmanian devil) and herbivores (giant panda, cow, horse, rabbit, and elephant). Only one embigin (*EMB*) gene had a convergent AAC in the carnivores (except Tasmanian devil) and there was no convergent AAC in the herbivores (Fig. 2b), congruent with the suggestion that adaptive molecular convergence linked to phenotypic convergence is rare [49]. Interestingly, *EMB*, which was predicted to be functionally altered in the three carnivore clades, is known to play a role in the outgrowth of motor neurons and in the formation of neuromuscular junctions [50]. We confirmed that the AAC in *EMB* gene is also conserved in the domestic ferret. Additionally, 18 and 56 genes were predicted to be carnivore-specific and herbivore-specific functions, respectively, altered by at least one AAC (Additional file 4: Datasheets S7 and S8). Among the carnivore-specific function altered genes, several genes are known to be associated with muscle contraction (*TMOD4* and *SYNC*) and steroid hormone synthesis (*STAR*).

### Family-wide highly conserved regions

**Fig. 3:**
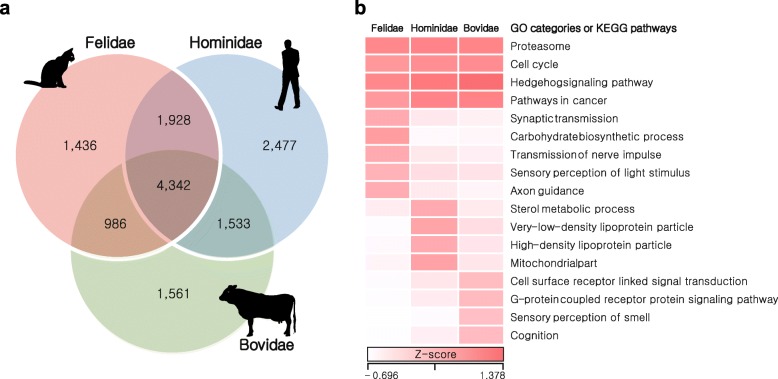
HCRs in Felidae, Hominidae, and Bovidae. HCRs in the same family species were identified by calculating the ratios between numbers of conserved and non-conserved positions. **a** *Venn diagrams* of genes in the HCRs. **b** *Heatmap* of enriched gene ontology (GO) categories or KEGG pathways in the HCRs. Z-scores for the average fractions of homozygous positions are shown as a *white-to-red color scale*

Conservation of DNA sequences across species reflects functional constraints and, therefore, characterizing genetic variation patterns is critical for understanding the dynamics of genomic change and relevant adaptation of each and a group of species [51, 52]. We scanned for homozygous genomic regions, which are strongly conserved among species within families: Felidae (cat, tiger, lion, cheetah, leopard, snow leopard, and leopard cat, divergence time: ~15.9 million years ago [MYA], carnivores), Hominidae (human, chimpanzee, bonobo, gorilla, and orangutan, ~15.8 MYA, omnivores), and Bovidae (cow, goat, sheep, water buffalo, and yak, ~26 MYA, herbivores) [53–55]. These highly conserved regions (HCRs) represent reduction in genetic variation (homozygous regions shared among species belonging to the same family; Fig. 3 and Additional file 3: Tables S39 and S42). A total of 1.13 Gb of Felidae, 0.93 Gb of Hominidae, and 0.88 Gb of Bovidae HCRs were detected with significantly reduced genetic variation (adjusted *P* < 0.0001, Fisher’s exact test corrected using the Benjamini–Hochberg method; Additional file 3: Table S43) compared with other genomic regions. A total of 4342 genes in the HCRs were shared in all three families and these genes were enriched in many key biological functions (cell cycle, pathways in cancer, proteasome, and Hedgehog signaling pathway; Fig. 3 and Additional file 3: Tables S44 and S45) as expected. We then investigated family-specific genes (1436 in Felidae, 2477 in Hominidae, and 1561 in Bovidae) in the HCRs. The Felidae-specific genes were significantly enriched in sensory perception of light stimulus (GO:0050953, 27 genes, *P* = 0.0022), synaptic transmission (GO:0007268, 33 genes, *P* = 0.0044), transmission of nerve impulse (GO:0019226, 37 genes, *P* = 0.0054), and axon guidance pathway (20 genes, *P* = 0.0054; Additional file 3: Tables S46 and S47), hinting to adaptation for the fast reflexes found in cats. Notably, the Felidae-specific genes were also functionally enriched for carbohydrate biosynthetic process (GO:0016051, 18 genes, *P* = 0.00061). This may be related to the predatory feeding pattern of felids (a meat-based diet, so low dietary availability of carbohydrates). On the other hand, the Bovidae-specific genes were enriched in sensory perception of smell (GO:0007608, 82 genes, *P* = 2.44 × 10^–16^) and cognition (GO:0050890, 113 genes, *P* = 2.54 × 10^–9^; Additional file 3: Tables S48–S50) functions, indicating herbivores’ adaptation for defense mechanisms from being poisoned by toxic plants [56].

### Genetic diversity and demographic history of Felidae species

**Fig. 4:**
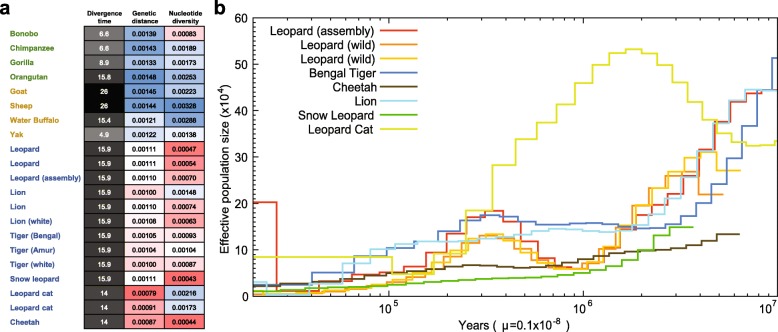
Genetic diversity in Felidae species. **a** Genetic distances and nucleotide diversities. Sequences of Felidae, Hominidae, and Bovidae were mapped to cat, human, and cow references, respectively. The genetic distances were calculated by dividing the number of homozygous SNVs to the reference genome by corresponding species genome size (bp) and divergence time (MYA). Nucleotide diversities were calculated by dividing the number of heterozygous SNVs by the genome size. The divergence times were from TimeTree database. **b** Estimated felids population sizes. Generation times of the leopard cat and big cats are three and five years, respectively. μ is mutation rate (per site, per year)

Carnivores tend to have smaller population sizes than species belonging to lower trophic groups, a characteristic argued to be associated with a higher propensity for extinction [1, 2]. We have investigated genetic diversity (which is affected by population size) in Felidae and compared it to different dietary requirement groups, omnivorous Hominidae and herbivorous Bovidae. The Felidae genetic diversity (0.00094 on average), based on the heterozygous single nucleotide variation (SNV) rates, is much lower than those of Hominidae (0.00175) and Bovidae (0.00244; Fig. 4a and Additional file 3: Tables S39 and S42). In terms of genomic similarity, Felidae showed the smallest genetic distances (0.00102 on average; see “Methods”), whereas larger genetic distances were detected in Hominidae (0.00141 on average) and Bovidae (0.00133 on average), suggesting that the extreme dietary specialization in the felids imposes strong and similar selection pressures on its members [1, 2]. The heterozygous SNV rates of leopards (0.00047–0.00070) are similar to those of snow leopard (0.00043), cheetah (0.00044), and white lion (0.00063), which have extremely low genetic diversity due to isolation or inbreeding [16, 19, 57], and smaller than those of lions (0.00074–0.00148) and tigers (0.00087–0.00104). The smaller cat (two leopard cats, 0.00173–0.00216) displays relatively high genetic diversity compared with the larger big cats, as previously reported [58]. Additionally, the demographic histories of felid species (leopards, tiger, cheetah, lion, snow leopard, and leopard cat) were constructed using a pairwise sequentially Markovian coalescent (PSMC) model inference [59]. The leopard cat showed a very different demographic history from the big cats: the population size of leopard cats increased between 10 million and 2 million years ago, whereas other big cats showed a consistent population decrease (Fig. 4b). It is predicted that the leopards experienced a severe genetic bottleneck between 2 million to 900 K years ago, whereas other big cats did not. The three leopard genomes showed a similar demographic history. However, over the last 30 K years, the assembled leopard genome showed an explosion in effective population size, whereas the wild leopards did not. The relatively large effective population size likely reflects that admixture occurred very recently between Amur leopard and North-Chinese leopard (*P. pardus japonensis*), as confirmed by the pedigree information (~30 % North-Chinese leopard admixture) and mitochondrial sequence analyses (Additional file 2: Figure S1), rather than an actual increase in population size. Cheetah and snow leopard showed low levels of effective population size in the last 3 million years, confirming their low genetic diversity [16, 19].

## Conclusions

Our study provides the first whole genome assembly of leopard which has the highest quality of big cat assembly reported so far, along with comparative evolutionary analyses with other felids and mammalian species. The comparative analyses among carnivores, omnivores, and herbivores revealed genetic signatures of adaptive convergence in carnivores. Unlike carnivores, omnivores and herbivores showed less common adaptive signatures, suggesting that there has been strong selection pressure for mammalian carnivore evolution [1, 2, 30]. The genetic signatures found in carnivores are likely associated with their strict carnivorous diet and lifestyle as an agile top predator. Therefore, cats are a good model for human diabetes study [29, 60, 61]. Our carnivore and Felidae analyses on diet-adapted evolution could provide crucial data resources to other human healthcare and disease research. At the same time, it is important to note that we focused on carnivores which specialize in consuming vertebrate meat. However, there are many different types of carnivores, such as insectivore (eating insects), invertivore (eating invertebrates), and hematophagy (consuming blood). Therefore, it is necessary to further investigate if the genetic signatures found in vertebrate meat eating carnivores are also shared in other carnivores and/or if the other carnivores show different patterns of evolutionary adaptation according to their major food types. Also, non-living or decaying material eating animals such as coprophagy (eating feces) and scavenger (eating carrion) could be a good subject for investigating evolutionary adaptations by diet patterns [62].

Felidae show a higher level of genomic similarity with each other when compared to Hominidae and Bovidae families, with a very low level of genetic diversity. While more detailed functional studies of all the selected candidate genes will be necessary to confirm the roles of individual genes, our comparative analysis of Felidae provides insights into carnivory-related genetic adaptations, such as extreme agility, muscle power, and specialized diet that make the leopards and Felidae such successful predators. These lifestyle-associated traits also make them genetically vulnerable, as reflected by their relatively low genetic diversity and small population sizes.

## Methods

### Sample and genome sequencing

A muscle sample was obtained from a dead female leopard acquired from the Daejeon O-World of Korea. The leopard sample was confirmed as ~30 % hybrid with North-Chinese leopard according to pedigree information. Phylogenetic analyses on mtDNA genes also confirmed that the leopard sample is a hybrid with North-Chinese leopard (Additional file 1: Supplemental Methods for details). We constructed 21 libraries with a variety of insert sizes (170 bp, 400 bp, 500 bp, 700 bp, 2 Kb, 5 Kb, 10 Kb, 15 Kb, and 20 Kb) according to the manufacturer’s protocol (Illumina, San Diego, CA, USA). The libraries were sequenced using Illumina HiSeq platforms (HiSeq2500 for short insert libraries and HiSeq2000 for long-mate pair libraries). We applied filtering criteria (polymerase chain reaction duplicated, adaptor contaminated, and < Q20 quality) to reduce the effects of sequencing errors in the assembly (Additional file 1: Supplemental Methods for details). The four wild Amur leopards (two for TSLRs and two for re-sequencing) and one Amur leopard cat samples, originated from Russia and Korea, respectively, were sequenced using HiSeq platforms.

### Genome assembly and annotation

The error corrected reads by *K*-mer analysis (*K* = 21) were used to assemble the leopard genome using SOAPdenovo2 software [21]. The short insert size libraries (<1 Kb) were assembled into distinct contigs based on the *K*-mer (*K* = 63) information. Read pairs from all the libraries then were used to scaffold the contigs step by step, from short to long insert size libraries. We closed the gaps using short insert size reads in two iterations. Only scaffolds exceeding 200 bp were used in this step. To reduce erroneous gap regions in the scaffolds, we aligned the ~0.8× Illumina TSLRs from the two wild Amur leopard individuals to the scaffolds using BWA-MEM [63] and corrected the gaps with the synthetic long reads using in-house scripts. Further details of the genome size estimation and genome assembly appear in the Supplemental Methods in Additional file 1. Assembly quality was assessed by mapping all of the paired-end DNA reads into the final scaffolds. The mapping was conducted using BWA-MEM. Also, the assembly and gene annotation qualities were assessed using BUSCO software [23].

The leopard genome was annotated for repetitive elements and protein-coding genes. For the repetitive elements annotation, we searched the leopard genome for tandem repeats and transposable elements, as previously described [16]. Detailed methods of the repetitive elements annotation are shown in the Supplemental Methods in Additional file 1. For the protein-coding gene prediction, homology-based gene prediction and *de novo* gene prediction were conducted. For the homology gene prediction, we searched for cat, tiger, dog, human, and mouse protein sequences from the NCBI database using TblastN (version 2.2.26) [64] with an *E*-value cutoff of 1E-5. The matched sequences were clustered using GenBlastA (version 1.0.4) [65] and filtered by coverage and identity of >40 % criterion. Gene models were predicted using Exonerate software (version 2.2.0) [66]. For the *de novo* gene prediction, AUGUSTUS (version 3.0.3) software [67] was used. We filtered out genes shorter than 50 amino acids, possible pseudogenes having premature stop-codons, and single exon genes that were likely to be derived from retro-transposition. Additionally, we annotated protein-coding genes of cheetah and lion genomes as their gene sets are preliminary.

### Comparative evolution analyses

Orthologous gene families were constructed for evolutionary analyses using OrthoMCL 2.0.9 software [68] with 17 mammalian genomes (seven carnivores: leopard, cat, tiger, cheetah, lion, polar bear, and killer whale; five omnivores: human, mouse, dog, pig, and opossum; and five herbivores: giant panda, cow, horse, rabbit, and elephant). Also, orthologous gene families were constructed with 18 mammalian genomes by adding Tasmanian devil for more taxonomically equivalent comparisons among the three different diet groups. Human, mouse, cat, tiger, dog, cow, pig, horse, elephant, rabbit, polar bear, giant panda, killer whale, opossum, and Tasmanian devil genomes and gene sets were downloaded from the NCBI database. To estimate divergence time of the mammalian species, we extracted only four-fold degenerate sites from the 18 mammalian single copy gene families using the CODEML program in PAML 4.5 package [38]. We estimate the divergence time among the 17 species (excepting Tasmanian devil in order to use only one out-group species) using the RelTime method [69]. The date of the node between human and opossum was constrained to 163.7 MYA, human–elephant was constrained to 105 MYA, and human–dog was constrained to 97.5 MYA according to divergence times from the TimeTree database [55]. The divergence times were calculated using the Maximum Likelihood method based on the Jukes–Cantor model [70]. The divergence time between out-group species (opossum and Tasmanian devil: 84.2 MYA) was obtained from the TimeTree database and directly used. The phylogenetic tree topology was derived from previous studies [71–74]. A gene expansion and contraction analysis was conducted using the CAFÉ program (version 3.1) [75] with the estimated phylogenetic tree information. We used the *P* < 0.05 criterion for significantly changed gene families.

To construct multiple sequence alignments among ortholog genes, PRANK [76] was used, and the CODEML program in PAML 4.5 was used to estimate the *d*_*N*_/*d*_*S*_ ratio (ω) [38]. The one-ratio model, which allows only a single *d*_*N*_/*d*_*S*_ ratio for all branches, was used to estimate the general selective pressure acting among all species. A free-ratios model was used to analyze the *d*_*N*_/*d*_*S*_ ratio along each branch. To further examine potential positive selection, the branch-site test of positive selection was conducted [39]. Statistical significance was assessed using likelihood ratio tests with a conservative 10 % FDR criterion [77]. We first performed this positive selection analysis for the 17 mammalian genomes (except Tasmanian devil). When we identified shared PSGs, genomes in the same diet group (carnivores, omnivores, and herbivores) were excluded from background species; for example, we excluded other carnivore genomes from the background species, when we identified PSGs of leopard. The PSGs of Tasmanian devil were separately identified, using Tasmanian devil as the foreground species and all of the omnivores and herbivores as background species, and then compared with the PSGs of the 17 mammalian species.

We also identified target species-specific AACs. To filter out biases derived from individual-specific variants, we used all of the Felidae re-sequencing data by mapping to the closest Felidae reference genome. The mapping was conducted using BWA-MEM, and variants were called using SAMtools-0.1.19 program [78] with the default options, except that the “-d 5 –D 200” option in the variants filter step was used. Function altering AACs were predicted using PolyPhen-2 [79] and PROVEAN v1.1 [80] with the default cutoff values. Human protein sequences were used as queries in this step. A convergent AAC was defined when all of the target species had the same amino acid in same sequence position. The carnivore-specific or herbivore-specific function altered genes were identified when all of the target species had at least one function altering AAC in any sequence position and all of the different diet species had no function altering AAC.

To characterize genetic variation in the genomes of three mammalian families (Felidae, Hominidae, and Bovidae), we scanned genomic regions that showed significantly reduced genetic variation by comparing variations of each window and whole genome (autosomes only). The Hominidae and Bovidae genome sequences were download from the NCBI database and were mapped to human (GRCh38) and cow (Bos_taurus_UMD_3.1.1) references, respectively. Variants (SNVs and indels) were called using SAMtools. The numbers of homozygous and heterozygous positions within each 100 Kb window (bin size = 100 Kb, step size = 10 Kb) were estimated by calculating the numbers of conserved and non-conserved bases in the same family genomes. We only used windows that covered more than 80 % of window size by all the mapped genomes. *P* values were calculated by performing Fisher’s exact test to test whether the ratio of homozygous to heterozygous positions in each window was significantly different from that of chromosomes. *P* values were corrected using the Benjamini–Hochberg method [81] and only adjusted *P* values of <0.0001 were considered significant. Only the middle 10 Kb of each significantly different window were considered as HCRs. For functional enrichment tests of candidate genes by all the comparative analyses, we used the DAVID bioinformatics resources [82].

### Genetic diversity and demographic history

The genetic distances were calculated by dividing the number of homozygous SNVs to the reference genome (the cat reference for Felidae, the human reference for Hominidae, and the cow reference for Bovidae genomes) by the corresponding species’ genome size (bp) and divergence time (MYA). Nucleotide diversities were calculated by dividing the number of heterozygous SNVs by the genome size.

Demographic histories of Felidae were analyzed using the PSMC program [59]. First, we aligned eight Felidae whole genome data (three leopards [one assembled and two re-sequenced], a Bengal tiger, a cheetah, a lion, a snow leopard, and a leopard cat) onto the Felis_catus_8.0 reference using BWA-MEM with default options. The consensus sequences of each Felidae genome were constructed using SAMtools software and then divided into non-overlapping 100 bp bins that were marked as homozygous or heterozygous on the basis of SNV datasets. The resultant bins were used as the input for demographic history analysis after removal of the sex chromosome parts. The demographic history of Felidae species was inferred using the PSMC model with -N25 -t15 -r5 -p “4 + 25*2 + 4 + 6” options, which have been used for great apes’ population history inference [83]. Bootstrapping was performed to determine the estimation accuracy by randomly resampling 100 sequences from the original sequences. The final results were plotted using a “psmc_plot.pl” script in PSMC utils with previously reported generation times (-g: three years for leopard cat, five years for big cats) and mutation rates (-u [per site, per year]: 1.1*e-9) [16, 84].

## Additional files

Additional file 1: Supplemental Methods. Further details of species identification using mtDNA consensus mapping method; raw read filtering criteria; genome size estimation using *K*-mer analysis; leopard genome assembly using various *K*-mer values; repeat annotation; species selection for comparative genomic analysis. (DOCX 43 kb)
Additional file 2: Figure S1. Species and sub-species identification for three leopard samples. **Figure S2.** Distribution of *K*-mer frequency in the error-corrected reads. **Figure S3.** GC content distributions. **Figure S4.** Composition of mammalian orthologous genes. **Figure S5.** Divergence time estimation of 18 mammals. **Figure S6.** Contraction of the amylase gene families (AMY1 and AMY2) in carnivores. **Figure S7.** Frame-shift mutations in Felidae *GCKR* genes. **Figure S8.** Felidae-specific amino acid changes in DNA repair system. **Figure S9.** Felidae-specific amino acid change in MEP1A protein. **Figure S10.** Felidae-specific amino acid change in ACE2 protein. **Figure S11.** Felidae-specific amino acid change in PRCP protein. (DOCX 2024 kb)
Additional file 3: Tables S1-50. (DOCX 174 kb)
Additional file 4: Datasheet S1. PSGs in leopard genome. Datasheet S2. Shared PSGs in Felidae. Datasheet S3. Shared PSGs in carnivores. Datasheet S4. Shared PSGs in omnivores. Datasheet S5. Shared PSGs in herbivores. Datasheet S6. Felidae-specific genes having function altering AACs. Datasheet S7. Carnivore-specific function altered genes. Datasheet S8. Herbivore-specific function altered genes. (XLS 632 kb)

## Abbreviations

AAC: Amino acid change
HCR: Highly conserved region
PSG: Positively selected gene
PSMC: Pairwise sequentially Markovian coalescent
SNV: Single nucleotide variation
TSLR: TruSeq synthetic long reads
