# Supplementary material for: Comparison of carnivore, omnivore, and herbivore mammalian genomes with a new leopard assembly
Source: Genome Biol. 2016 Oct 11;17:211. doi: 10.1186/s13059-016-1071-4 (PMC5090899; doi:10.1186/s13059-016-1071-4)
Supplement: Additional file 1: — Supplemental Methods. Further details of species identification using mtDNA consensus mapping method; raw read filtering criteria; genome size estimation using K-mer analysis; leopard genome assembly using various K-mer values; repeat annotation; species selection for comparative genomic analysis. (DOCX 43 kb) [file 13059_2016_1071_MOESM1_ESM.docx]

**Supplemental Methods**

**Species identification using mtDNA consensus mapping method**

An Amur leopard individual for *de novo* assembly was known to contain ~30% North-Chinese leopard admixture according to the pedigree information. To confirm its sub-species, DNA reads from three Amur leopard genomes (one for *de novo* assembly and two for re-sequencing) were aligned to previously reported mitochondrial sequences of *Panthera pardus* (accession: EF551002.1) using BWA-MEM [1] with default options. Variants were called using the mpileup command in SAMtools [2], and consensus mitochondrial sequences of the three Amur leopards were generated using the vcf2fq command. Because only two gene (*NADH5* and *CYTB*) sequences for Amur leopard (*Panthera pardus orientalis*) are available, we compared the consensus mitochondrial sequences with known gene sequences of *Panthera*.

**Raw read filtering criteria**

To reduce sequencing error effects in assembling the reference leopard genome, we filtered out low quality or contaminated reads (both short insert libraries and long-mate pair libraries). The filtering criteria for exclusion were as follows:

1. PCR duplications (reads were considered duplicates when read1 and read2 of the two paired end reads were identical)
2. Reads with sequencing adapter contamination

Sequencing adapter left= "*GATCGGAAGAGCACACGTCTGAACTCCAGTCAC*"

Sequencing adapter right= "*GATCGGAAGAGCGTCGTGTAGGGAAAGAGTGT*"

1. Reads with ambiguous base (N) for more than 5% of the reads
2. Reads with an average base quality below 20 (<Q20)
3. Reads with junction adapter contamination for mate-pair libraries

Junction adapter left = "*CTGTCTCTTATACACATCT*"

Junction adapter right = "*AGATGTGTATAAGAGACAG*"

1. 2bp of 5’-end and 8bp of 3’-end of each read from short insert libraries were trimmed to filter out low-quality read ends

For additional re-sequenced genomes (two wild Amur leopards and one Amur leopard cat), we filtered out reads if the reads’ Q20 base content was lower than 70 %.

**Genome size estimation using *K*-mer analysis**

Prior to the estimation of leopard genome size, the sequence errors in the filtered reads were corrected based on *K*-mer frequency (*K*=21) information using Corrector_HA command of SOAPec program in SOAPdenovo2 package [3] with a 3-depth criterion for low-frequency *K*-mer cutoff. Then, we estimated the leopard genome size by *K*-mer analysis (*K*=21, 31, and 61) using JELLYFISH program [4]. The genome size was calculated by dividing the total number of *K*-mers by a peak depth of *K*-mers. The size of leopard genome was estimated to be approximately 2.45 Gb.

**Leopard genome assembly using various *K*-mer values**

The error corrected reads by *K*-mer analysis (*K*=21) were used to the leopard genome assembly using SOAPdenovo2 which employs the *de Bruijn* graph algorithm. As the quality of assembled genome can be affected by the *K*-mer size, we first assembled the error-corrected reads from short insert size libraries (<1 Kb) into distinct contigs with various *K*-mer values (*K*=27, 37, 43, 47, 53, 57, 63, 67, 73, and 77) using ‘pregraph’ and ‘contig’ commands in SOAPdenovo2 package. By considering assembled contig and N50 lengths, we chose contig sets from *K*=63, 67, 73, and 77 for further scaffolding analyses.

Prior to scaffolding, the insert size distributions of all the libraries were determined by aligning them onto the assembled contigs. We performed scaffolding the contig sets with read pairs from all the libraries step by step, from short to long insert size libraries using ‘map’ and ‘scaff’ commands in SOAPdenovo2 package. The estimated mode values of the insert size distributions and their upper and lower 20% boundaries were used in this step. By considering its assembled sequence and N50 lengths, we finally used scaffolds from *K*=63 for further analyses. We closed the gaps in scaffolds with short insert size reads in two iterations using GapCloser in SOAPdenovo2 package.

Next, we aligned the reads from short insert size libraries onto the scaffolds using BWA-MEM with default options. SAMtools was used to identify variants. Homozygous variants and heterozygous variants that were different from the assembled sequences were used to correct possible erroneous sequences in the scaffolds.

To reduce erroneous gap regions in the scaffolds, we also aligned the Illumina TSLRs from the two wild Amur leopard individuals to the scaffolds using BWA-MEM, and corrected the gaps with the synthetic long reads using in-house scripts.

Assembly quality was assessed by mapping all of the paired-end reads onto the final scaffolds using BWA-MEM. The assembly and gene annotation qualities were also assessed using BUSCO software [5].

**Repeat annotation**

For the annotation of repetitive elements for the assembled leopard genome, we searched the genome for tandem repeats using the Tandem Repeats Finder (version 4.07b) [6]. Transposable elements (TEs) were identified in the genome by homology-based and *ab initio*-based approaches. The homology-based approach was used with Repbase [7] database version 19.03 to identify repeats using RepeatMasker (version 4.0.5) [8] and RMBlast version 2.2.28 [9]. For *ab initio*-based approach, we used RepeatModeler version 1.0.7 [10]. All predicted repetitive elements were merged for statistics by in-house scripts.

**Species selection for comparative genomic analysis**

For comparative evolutionary analyses of three diet groups (carnivores, omnivores, and herbivores), we selected a total of 18 mammalian genomes by considering the following criteria:

1. Availability from genome sequences and gene set from NCBI databases
2. Assembly quality (N50 length of assembled fragments) and the number of predicted genes
3. Scientific classification (order, family, and genus) and divergence times to related species
4. Species that is closely related to other species having different diet patterns

(e.g., domestic dog, giant panda, and polar bear in Carnivora order; pig, cow, and killer whale in Cetartiodactyla clade)

1. Species having a strict diet pattern. For carnivores, only meat-eating carnivorous animals were considered. Tasmanian devil is a meat-eating marsupial carnivore, but it is also a well-known scavenger [11]. We included Tasmanian devil in our analyses for more taxonomically equivalent comparisons.
2. Leopard (from this study), cat, tiger, lion, and cheetah genomes were included to focus analyses on Felidae species

Finally, eight carnivorous (leopard, cat, tiger, cheetah, lion, polar bear, killer whale, and Tasmanian devil), five omnivorous (human, mouse, dog, pig, and opossum), and five herbivorous (giant panda, cow, horse, rabbit, and elephant) mammalian genomes were selected for the comparative genomic analyses.

**Supplemental References**

1. Li H. Aligning sequence reads, clone sequences and assembly contigs with BWA-MEM. eprint ArXiv. 2013;1303:3997.

2. Li H, Handsaker B, Wysoker A, Fennell T, Ruan J, Homer N, et al. The Sequence Alignment/Map format and SAMtools. Bioinformatics. 2009;25:2078–9.

3. Luo R, Liu B, Xie Y, Li Z, Huang W, Yuan J, et al. SOAPdenovo2: an empirically improved memory-efficient short-read de novo assembler. Gigascience. 2012;1:18.

4. Marçais G, Kingsford C. A fast, lock-free approach for efficient parallel counting of occurrences of k-mers. Bioinformatics. 2011;27:764–70.

5. Simão FA, Waterhouse RM., Ioannidis P, Kriventseva EV, Zdobnov EM. BUSCO: assessing genome assembly and annotation completeness with single-copy orthologs. Bioinformatics. 2015;31:3210-2.

6. Benson G. Tandem repeats finder: a program to analyze DNA sequences. Nucleic Acids Res. 1999;27:573–80.

7. Jurka J, Kapitonov VV, Pavlicek A, Klonowski P, Kohany O, Walichiewicz J, et al. Repbase Update, a database of eukaryotic repetitive elements. Cytogenet Genome Res. 2005;110:462–7.

8. Bedell JA, Korf I, Gish W. MaskerAid: a performance enhancement to RepeatMasker. Bioinformatics. 2000;16:1040–1.

9. RMBlast. http://www.repeatmasker.org/RMBlast.html. Accessed 16th Aug 2016.

10. Abrusán G, Grundmann N, DeMester L, Makalowski W. TEclass—a tool for automated classification of unknown eukaryotic transposable elements. Bioinformatics. 2009;25:1329–30.

11. Owen D, Pemberton D. Tasmanian Devil: A Unique and Threatened Animal. Sydney: Allen & Unwin; 2005.
