# Supplementary material for: Comparison of carnivore, omnivore, and herbivore mammalian genomes with a new leopard assembly
Source: Genome Biol. 2016 Oct 11;17:211. doi: 10.1186/s13059-016-1071-4 (PMC5090899; doi:10.1186/s13059-016-1071-4)
Supplement: Additional file 2: Figure S1. — Species and sub-species identification for three leopard samples. Figure S2. Distribution of K-mer frequency in the error-corrected reads. Figure S3. GC content distributions. Figure S4. Composition of mammalian orthologous genes. Figure S5. Divergence time estimation of 18 mammals. Figure S6. Contraction of the amylase gene families (AMY1 and AMY2) in carnivores. Figure S7. Frame-shift mutations in Felidae GCKR genes. Figure S8. Felidae-specific amino acid changes in DNA repair system. Figure S9. Felidae-specific amino acid change in MEP1A protein. Figure S10. Felidae-specific amino acid change in ACE2 protein. Figure S11. Felidae-specific amino acid change in PRCP protein. (DOCX 2024 kb) [file 13059_2016_1071_MOESM2_ESM.docx]

**Additional file 2**

A. *NADH5* gene


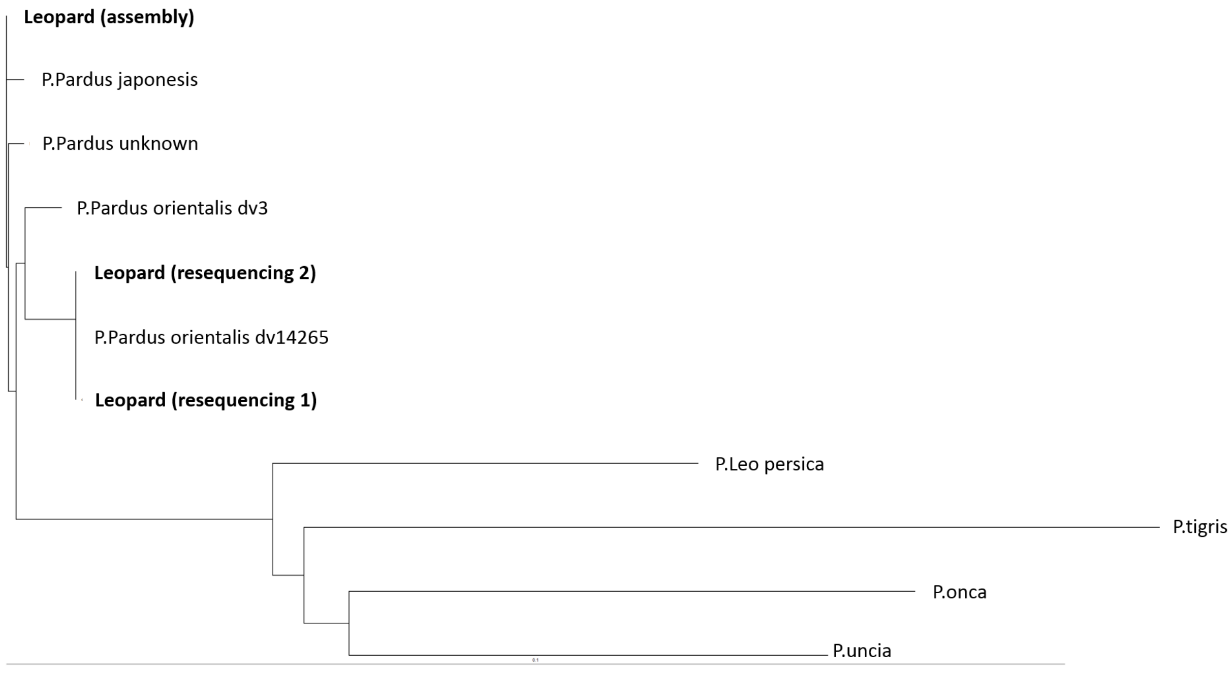


B. *CYTB gene*


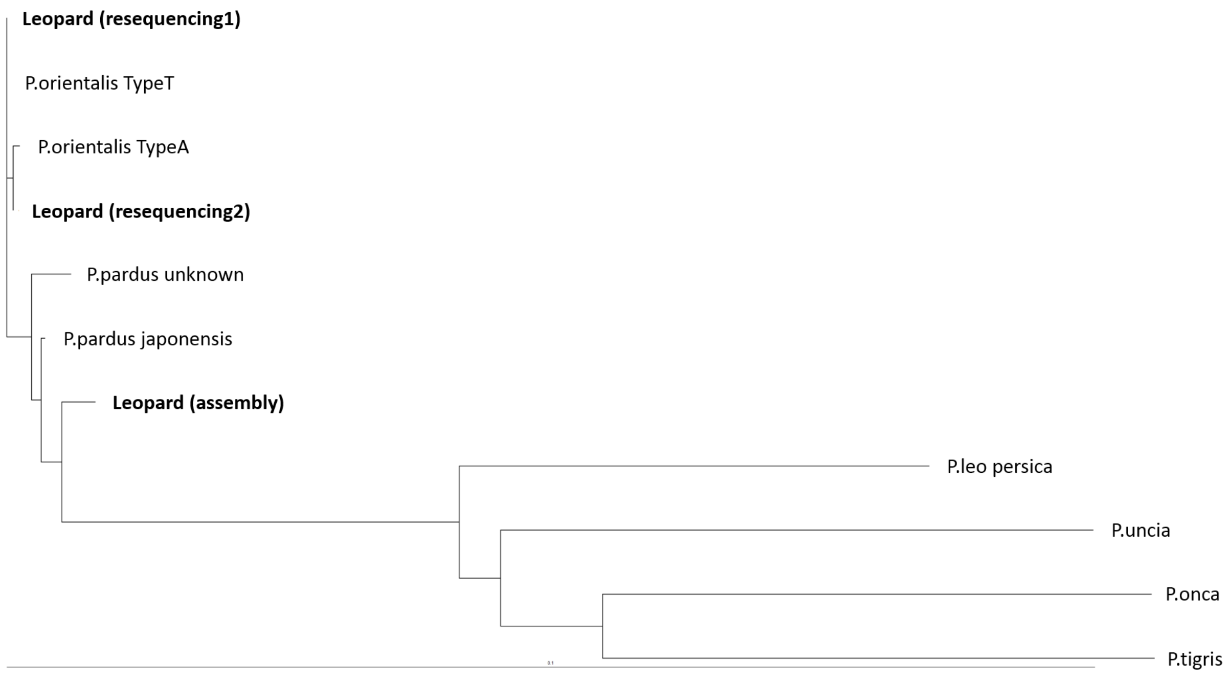


**Figure S1. Species and sub-species identification for three leopard samples.** (A) *NADH5* and (B) *CYTB* sequences for the three leopards were generated by mapping their reads to the previously reported mitochondrial sequences of *Panthera pardus* (Accession: EF551002.1).


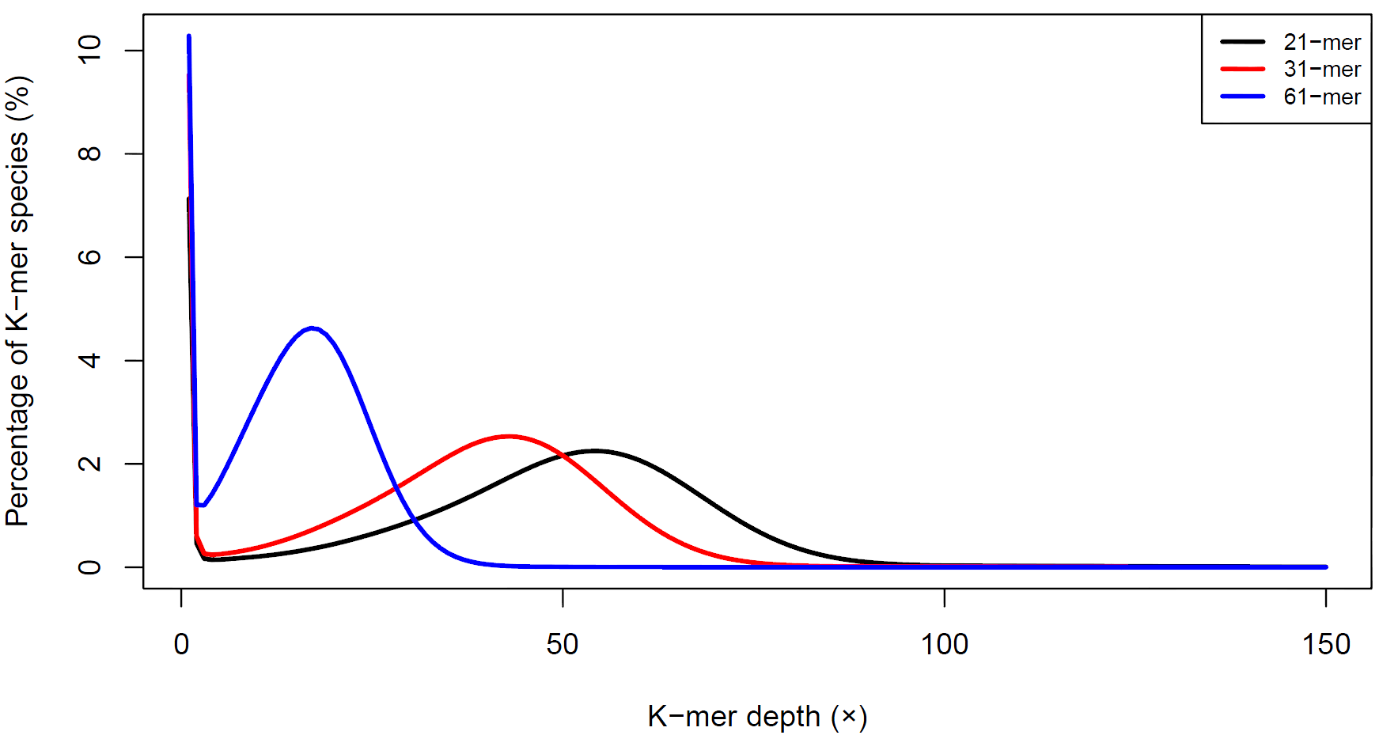


**Figure S2. Distribution of *K*-mer frequency in the error-corrected reads.** The x-axis represents depth, and the y-axis represents proportion, as calculated by the frequency at that depth divided by the total frequency at all depths.

**
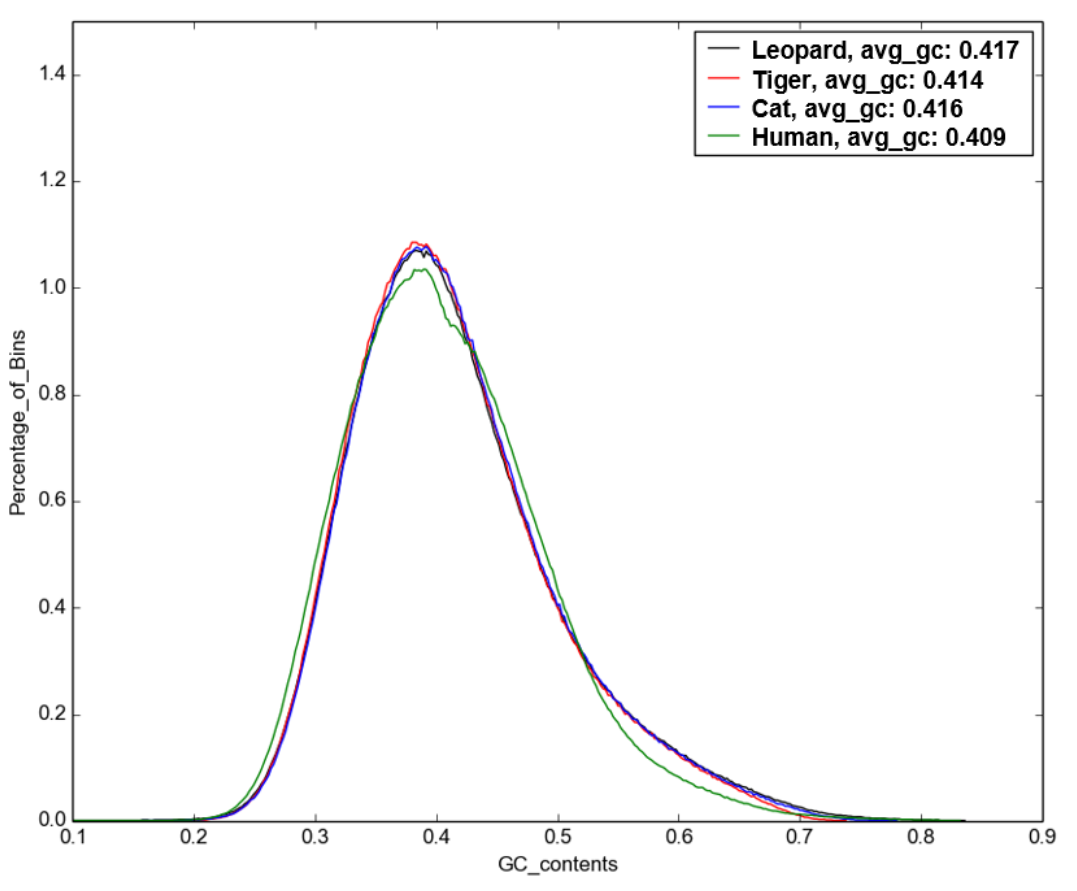
 Figure S3. GC content distributions.** The x-axis is GC content and the y-axis is the proportion of the bin number with the specified GC content.


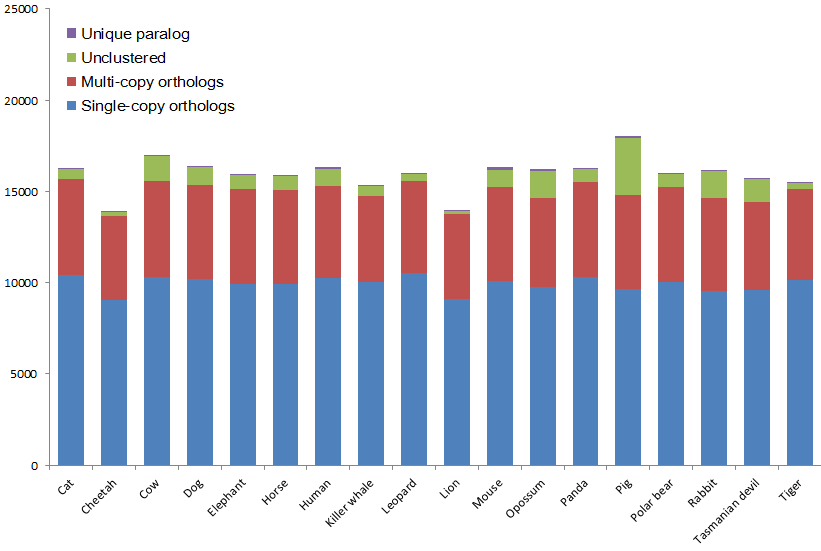


**Figure S4. Composition of mammalian orthologous genes.** A comparative representation of orthologous and paralogous genes in 18 mammalian genomes are shown.


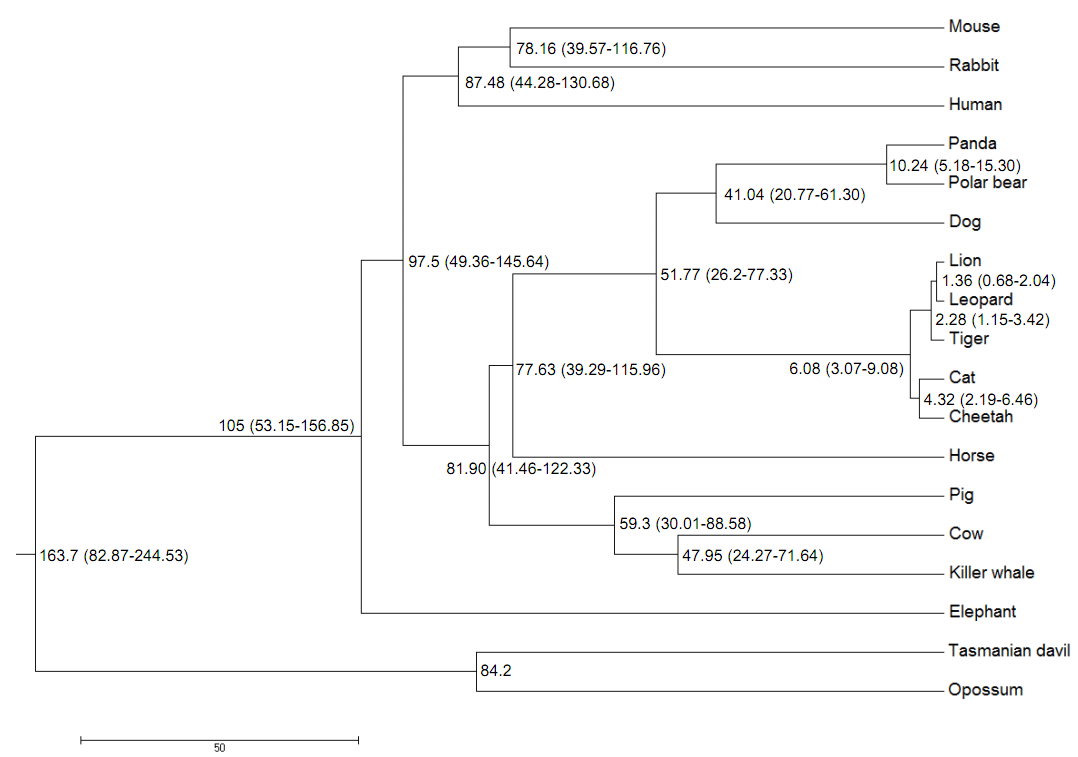


**Figure S5. Divergence time estimation of 18 mammals.** The divergence time from present (million years ago; MYA) is given at the nodes, with the 95% confidence intervals in parentheses. The calibration times of opossum-human (163.7 MYA), human-elephant (105 MYA) and human-dog (97.5 MYA) divergence were derived from the TimeTree database. The divergence time between out-group species (opossum and Tasmanian devil: 84.2 MYA) was obtained from the database and directly used. The topology was derived from previous studies.


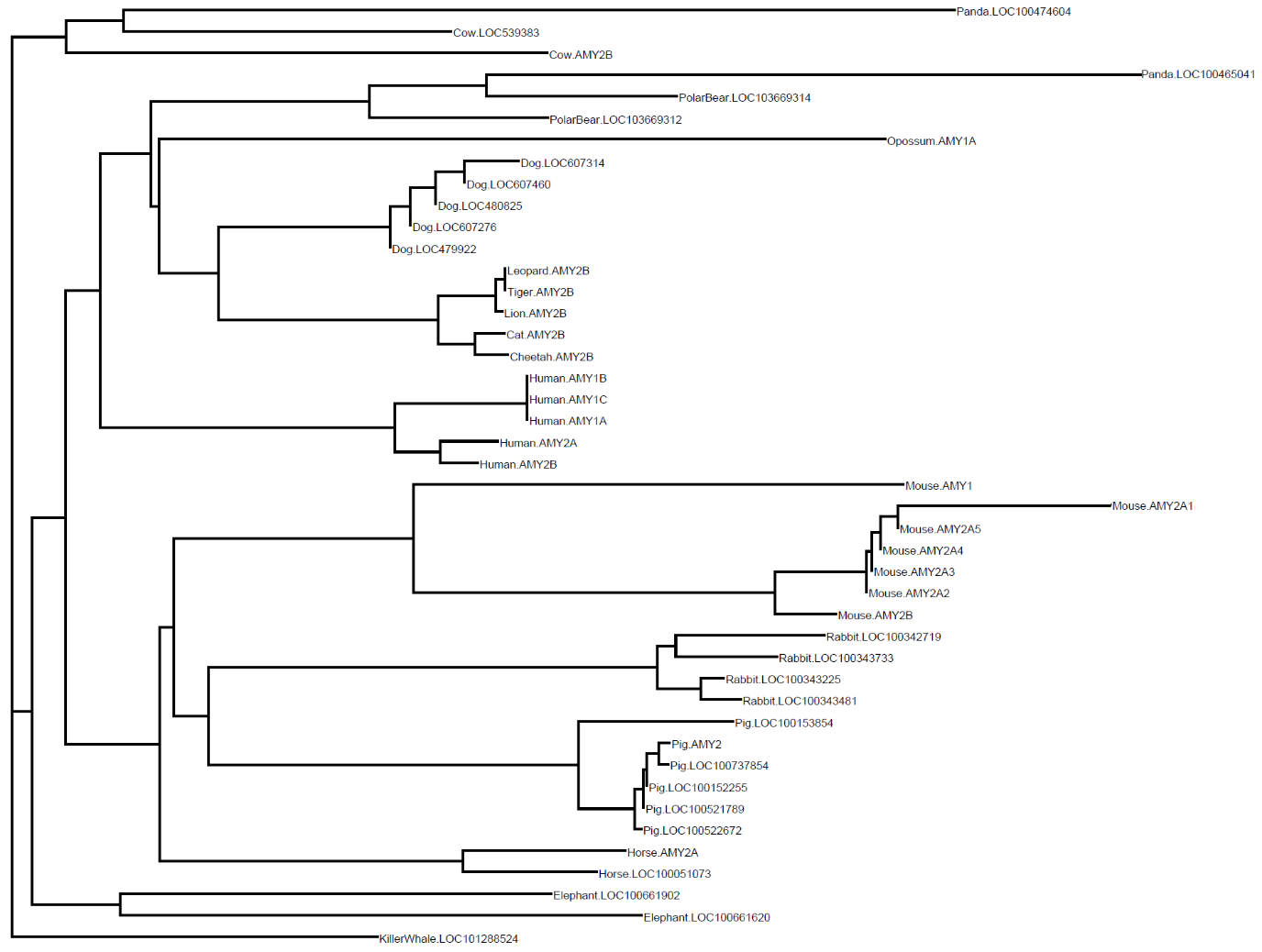


**Figure S6. Contraction of the amylase gene families (AMY1 and AMY2) in carnivores.** The amylase gene sequences were derived from contracted orthologous gene families in the eight carnivores (leopard, tiger, lion, cheetah, cat, polar bear, killer whale, and Tasmanian devil).


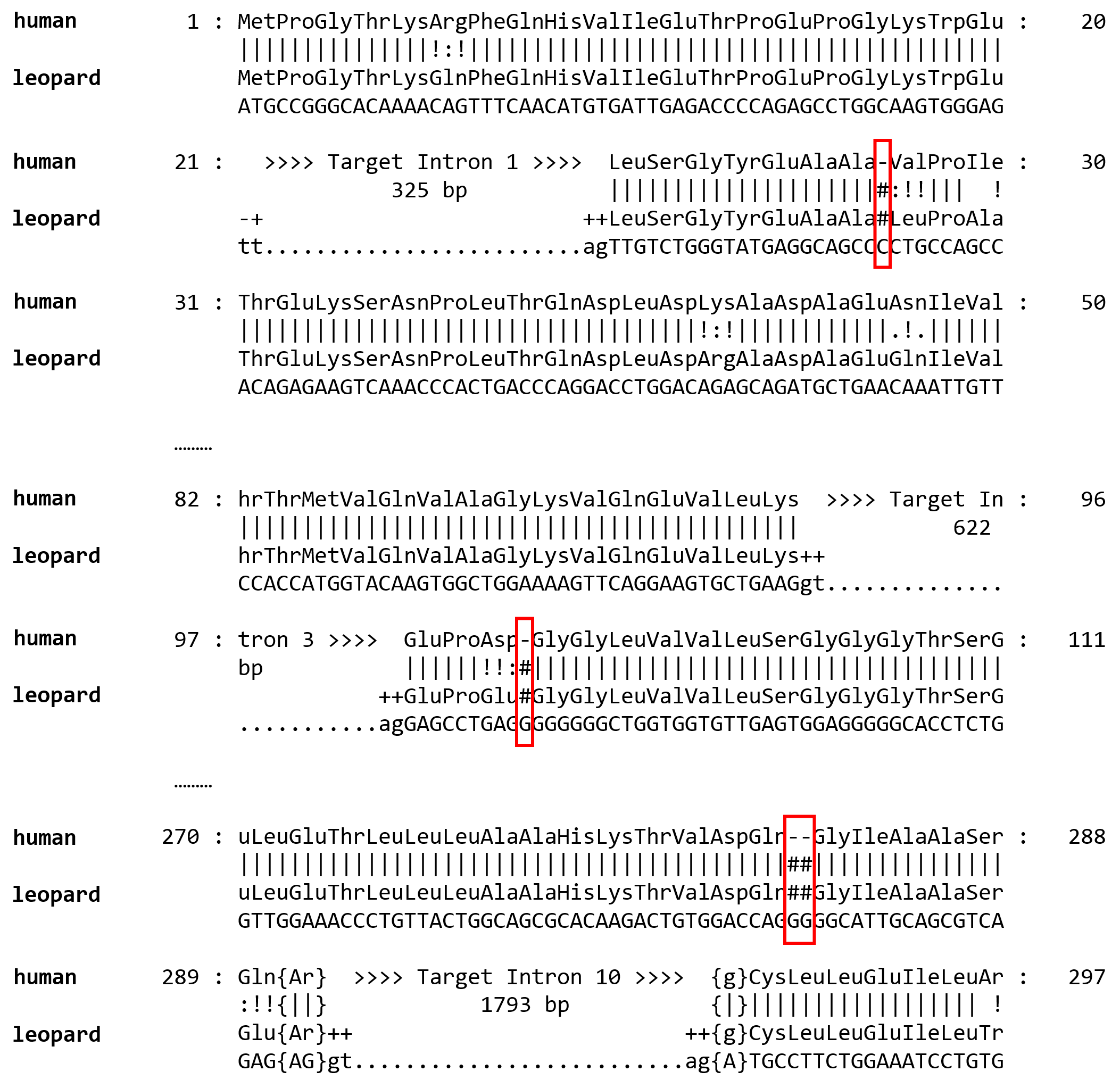


**Figure S7. Frame-shift mutations in Felidae *GCKR* genes.** The Felidae *GCKR* gene is pseudogenized by frame-shift mutations (red rectangles). Leopard, cat, tiger, lion, and snow leopard had a frame-shift mutation in exons 2, 4, and 10, respectively. Cheetah had a frame-shift mutation in exons 2 and 10. Leopard cat had a frame-shift mutation in exons 2 and 4. Only the protein sequence alignment of human and leopard is shown in this figure.


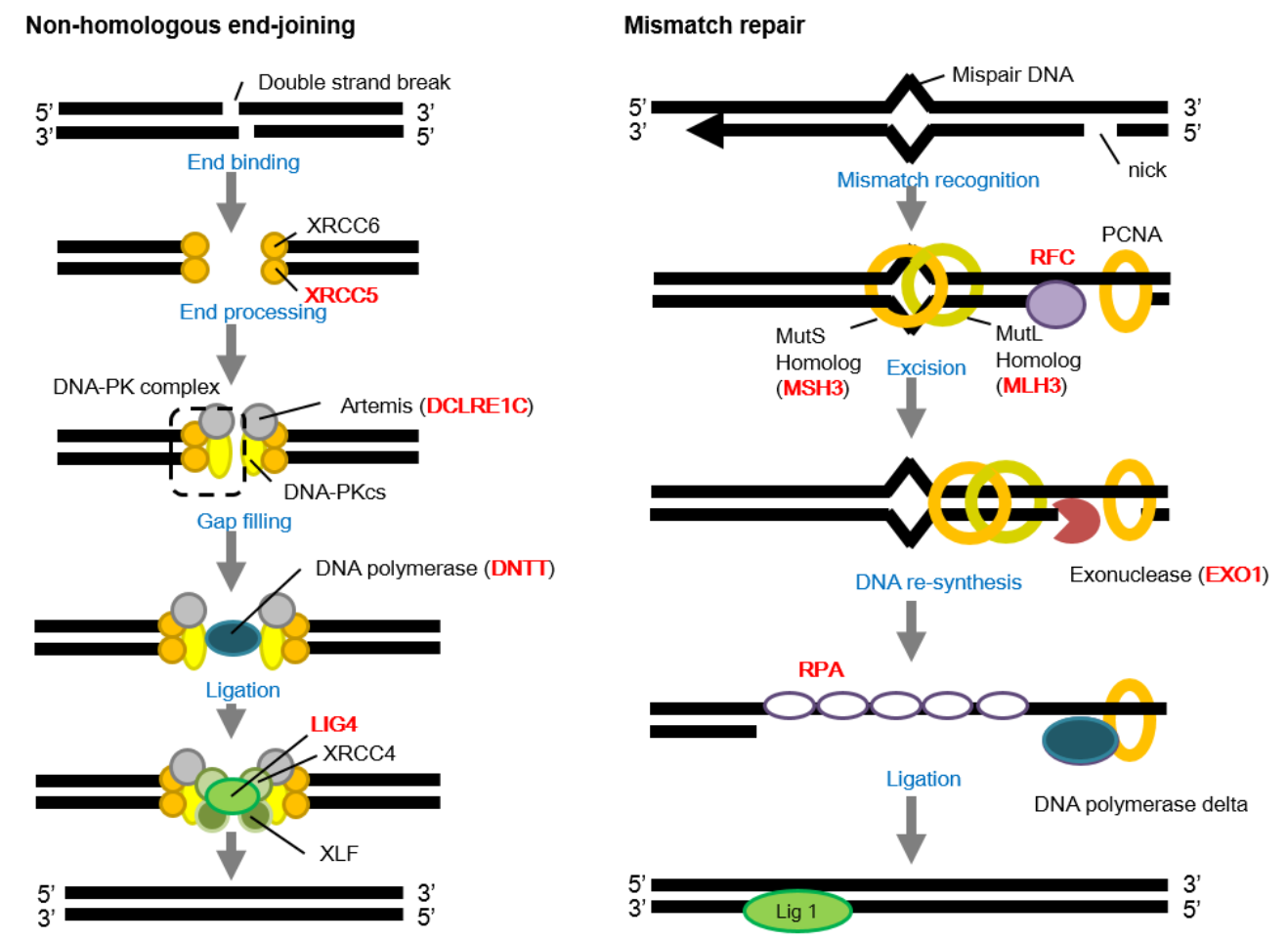


**Figure S8. Felidae-specific amino acid changes in DNA repair system.** Genes with Felidae-specific function altering amino acid changes in the non-homologous end-joining (KEGG pathway map03450) and mismatch repair (map03430) pathways are shown in red.


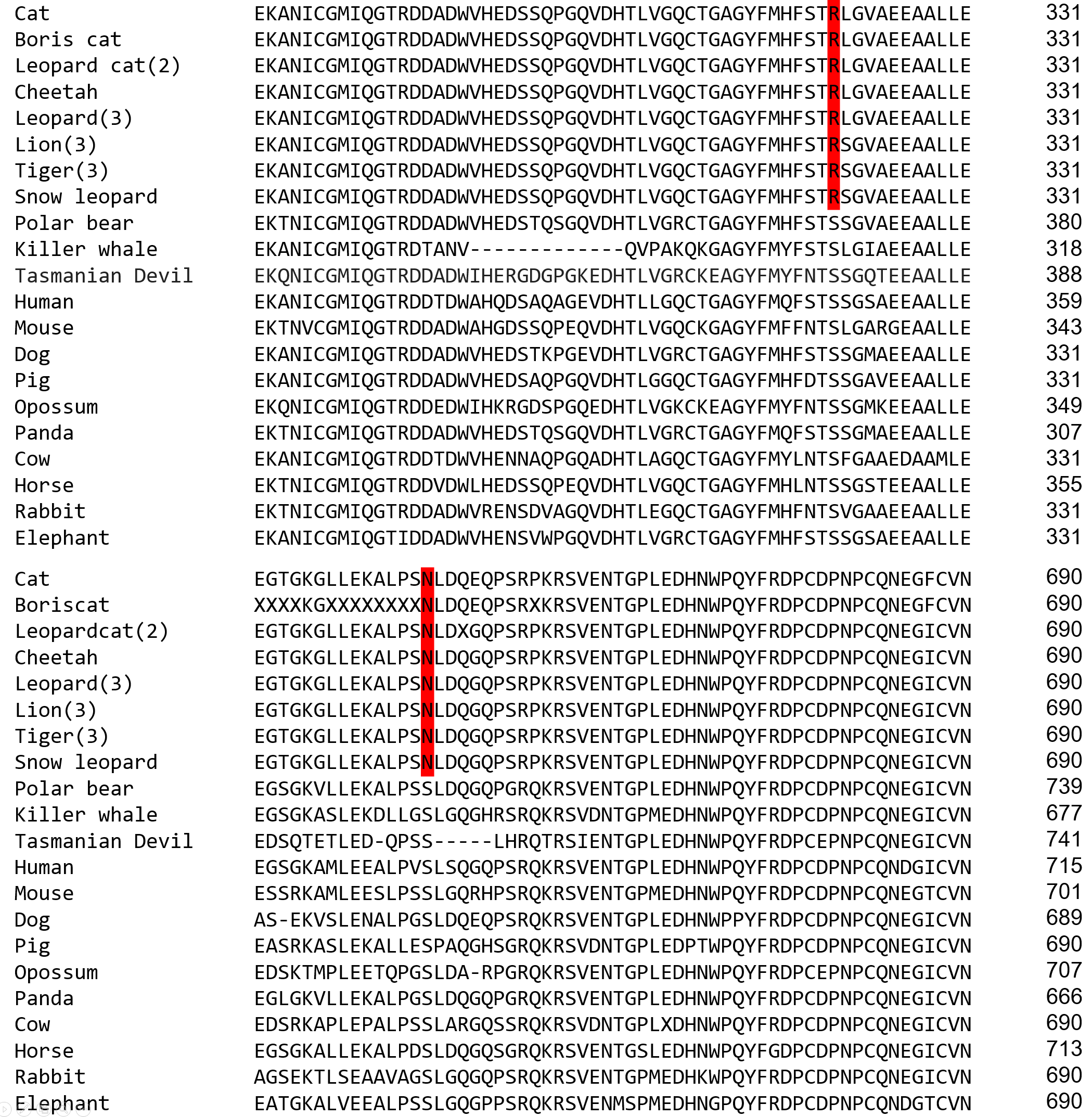


**Figure S9. Felidae-specific amino acid change in MEP1A protein.** Red rectangles indicate Felidae (2 cats, 2 leopard cats, 1 cheetah, 3 leopards, 3 lions, 3 tigers, and 1 snow leopard)-specific amino acid changes.

**
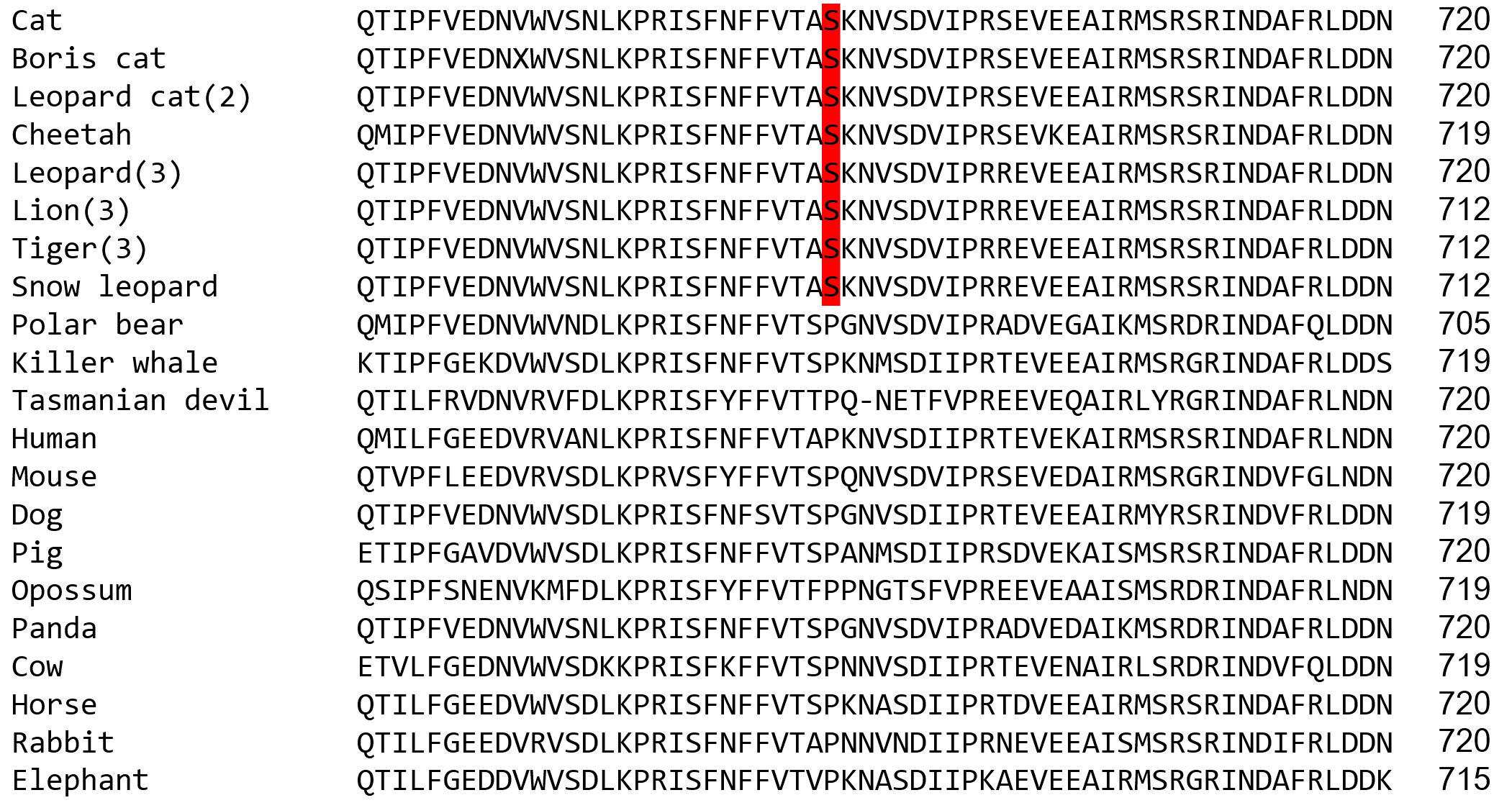
**

**Figure S10. Felidae-specific amino acid change in ACE2 protein.** Red rectangle indicates Felidae (2 cats, 2 leopard cats, 1 cheetah, 3 leopards, 3 lions, 3 tigers, and 1 snow leopard)-specific amino acid changes.

**
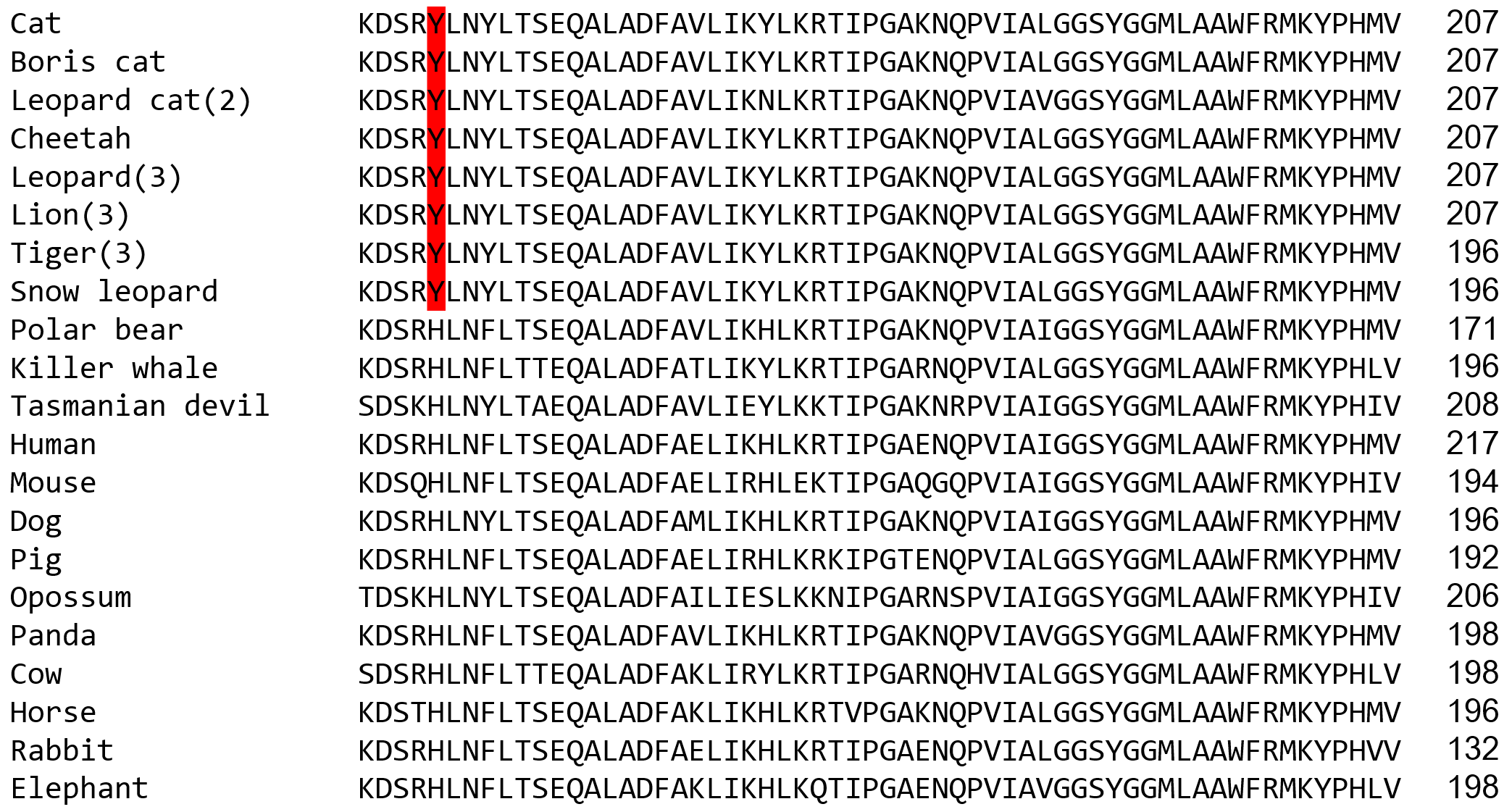
**

**Figure S11. Felidae-specific amino acid change in PRCP protein.** Red rectangle indicates Felidae (2 cats, 2 leopard cats, 1 cheetah, 3 leopards, 3 lions, 3 tigers, and 1 snow leopard)-specific amino acid changes.
