# Supplementary material for: Comparison of carnivore, omnivore, and herbivore mammalian genomes with a new leopard assembly
Source: Genome Biol. 2016 Oct 11;17:211. doi: 10.1186/s13059-016-1071-4 (PMC5090899; doi:10.1186/s13059-016-1071-4)
Supplement: Additional file 3: — Tables S1-50. (DOCX 174 kb) [file 13059_2016_1071_MOESM3_ESM.docx]

**Additional file 3**

**Table S1. Information regarding the sequencing libraries used for the leopard genome assembly**

| Insert size | Library | Number of read pairs | Read length (bp) | Total bases (bp) | Depth (genome size: 2.4Gb) | Depth sum  (×) |
| --- | --- | --- | --- | --- | --- | --- |
|  |  |  |  |  |  |  |
| 170bp | L1 | 333,553,833 | 100 | 66,710,766,600 | 27.8 | 55.4 |
|  | L2 | 331,689,208 | 100 | 66,337,841,600 | 27.6 |  |
| 400bp | L1 | 509,606,350 | 100 | 101,921,270,000 | 42.5 | 42.5 |
| 500bp | L1 | 186,089,810 | 100 | 37,217,962,000 | 15.5 | 15.5 |
| 700bp | L1 | 263,028,961 | 100 | 52,605,792,200 | 21.9 | 43.0 |
|  | L2 | 253,491,380 | 100 | 50,698,276,000 | 21.1 |  |
| 2kb | L1 | 253,118,942 | 50 | 25,311,894,200 | 10.5 | 33.0 |
|  | L2 | 276,971,258 | 50 | 27,697,125,800 | 11.5 |  |
|  | L3 | 261,524,182 | 50 | 26,152,418,200 | 10.9 |  |
| 5kb | L1 | 281,035,858 | 50 | 28,103,585,800 | 11.7 | 34.4 |
|  | L2 | 287,435,970 | 50 | 28,743,597,000 | 12.0 |  |
|  | L3 | 257,155,971 | 50 | 25,715,597,100 | 10.7 |  |
| 10kb | L1 | 258,914,666 | 50 | 25,891,466,600 | 10.8 | 30.5 |
|  | L2 | 259,983,330 | 50 | 25,998,333,000 | 10.8 |  |
|  | L3 | 212,309,118 | 50 | 21,230,911,800 | 8.8 |  |
| 15kb | L1 | 265,550,235 | 50 | 26,555,023,500 | 11.1 | 35.1 |
|  | L2 | 268,908,501 | 50 | 26,890,850,100 | 11.2 |  |
|  | L3 | 152,780,961 | 51 | 15,583,658,022 | 6.5 |  |
|  | L4 | 150,132,379 | 51 | 15,313,502,658 | 6.4 |  |
| 20kb | L1 | 266,551,535 | 50 | 26,655,153,500 | 11.1 | 20.8 |
|  | L2 | 231,700,291 | 50 | 23,170,029,100 | 9.7 |  |
| Total | - | 5,561,532,739 | - | 744,505,054,780 | 310.2 | |

**Table S2. Filtered sequence information**

| Library | | Number of raw read pairs | Number of remained read pairs | Trimmed read length | Remained total bases (bp) | Remained sequence depth (×) |
| --- | --- | --- | --- | --- | --- | --- |
|  |  |  |  |  |  |  |
| 170bp | L1 | 333,553,833 | 324,819,579 | 90 | 58,467,524,220 | 24.4 |
|  | L2 | 331,689,208 | 322,720,798 | 90 | 58,089,743,640 | 24.2 |
| 400bp | L1 | 509,606,350 | 463,815,627 | 90 | 83,486,812,860 | 34.8 |
| 500bp | L1 | 186,089,810 | 177,877,901 | 90 | 32,018,022,180 | 13.3 |
| 700bp | L1 | 263,028,961 | 247,339,040 | 90 | 44,521,027,200 | 18.6 |
|  | L2 | 253,491,380 | 233,469,831 | 90 | 42,024,569,580 | 17.5 |
| 2kb | L1 | 253,118,942 | 70,512,242 | 50 | 7,051,224,200 | 2.9 |
|  | L2 | 276,971,258 | 78,840,634 | 50 | 7,884,063,400 | 3.3 |
|  | L3 | 261,524,182 | 82,556,740 | 50 | 8,255,674,000 | 3.4 |
| 5kb | L1 | 281,035,858 | 46,062,964 | 50 | 4,606,296,400 | 1.9 |
|  | L2 | 287,435,970 | 55,322,387 | 50 | 5,532,238,700 | 2.3 |
|  | L3 | 257,155,971 | 55,745,264 | 50 | 5,574,526,400 | 2.3 |
| 10kb | L1 | 258,914,666 | 44,225,626 | 50 | 4,422,562,600 | 1.8 |
|  | L2 | 259,983,330 | 35,628,557 | 50 | 3,562,855,700 | 1.5 |
|  | L3 | 212,309,118 | 38,425,313 | 50 | 3,842,531,300 | 1.6 |
| 15kb | L1 | 265,550,235 | 25,137,484 | 50 | 2,513,748,400 | 1.0 |
|  | L2 | 268,908,501 | 23,451,001 | 50 | 2,345,100,100 | 1.0 |
|  | L3 | 152,780,961 | 9,374,114 | 51 | 956,159,628 | 0.4 |
|  | L4 | 150,132,379 | 6,094,053 | 51 | 621,593,406 | 0.3 |
| 20kb | L1 | 266,551,535 | 23,636,971 | 50 | 2,363,697,100 | 1.0 |
|  | L2 | 231,700,291 | 24,209,031 | 50 | 2,420,903,100 | 1.0 |
| Total | - | 5,561,532,739 | 2,389,265,157 | - | 158.6 | |

**Table S3. Estimation of leopard genome size based on *K*-mer frequency in the error-corrected reads**

| *K*-mer size | *K*-mer total num | *K*_depth of peak | Estimated genome size |
| --- | --- | --- | --- |
| 21 | 116,054,812,460 | 54 | 2,149,163,194 |
| 31 | 95,405,247,995 | 43 | 2,218,726,698 |
| 61 | 41,733,211,831 | 17 | 2,454,894,814 |

**Table S4. The leopard genome assembly statistics**

|  | Contig | | Scaffold | |
| --- | --- | --- | --- | --- |
|  | Size (bp) | Number | Size (bp) | Number |
| N90 | 5,500 | 122,036 | 3,467,308 | 135 |
| N80 | 9,132 | 87,540 | 8,979,855 | 93 |
| N70 | 12,732 | 64,613 | 12,770,773 | 68 |
| N60 | 16,634 | 47,584 | 18,513,618 | 51 |
| N50 | 20,993 | 34,310 | 21,701,857 | 39 |
| Longest | 240,914 | ----- | 84,051,066 | ----- |
| Total Size | 2,478,888,723 | ----- | 2,578,022,254 | ----- |
| Total Number  (>100bp) | ----- | 265,235 | ----- | 50,400 |
| Total Number  (>2Kb) | ----- | 174,791 | ----- | 2,670 |

**Table S5. Sample information of wild Amur leopards and Amur leopard cat used in the present study**

| Species | Sequence | ID | Gender | Data collection | Origin |
| --- | --- | --- | --- | --- | --- |
| *Panthera pardus orientalis* | HiSeq2500 | PPO1 | M | 29 Oct 2006 | Nezhenka (Sanduga) river basin , Nadezhdensky Region, Primorsky Krai |
| *Panthera pardus orientalis* | TSLR | PPO2 | M | 02 Nov 2006 | Nezhenka (Sanduga) river basin , Nadezhdensky Region, Primorsky Krai |
| *Panthera pardus orientalis* | TSLR | PPO4 | F | 15 Oct 2007 | Malaya Ananievka (Elduga) river basin, Nadezhdensky Region, Primorsky Krai |
| *Panthera pardus orientalis* | HiSeq2500 | PPO5 | unknown | 18 Oct 2008 | Bolshaya Ananievka (Elduga) river basin , Nadezhdensky Region, Primorsky Krai |
| *Prionailurus bengalensis*  *euptilurus* | HiSeq2500 | - | M | N/A | Republic of Korea |

**Table S6. Illumina TruSeq Synthetic Long Reads from two wild Amur leopard individuals**

| # Sequences | 393,866 |
| --- | --- |
| Total bases (bp) | 1,999,851,886 |
| Average length (bp) | 5,077 |
| Standard deviation (bp) | 3,311 |
| The longest length (bp) | 21,607 |
| The shortest length (bp) | 1,000 |
| N50 (bp) | 8,293 |
| GC contents | 40.18% |
| N bases | 0.00% |

**Table S7. Statistics regarding predicted protein-coding genes**

| Gene set | | Number | Average  transcript length (bp) | Average CDS length (bp) | Average no. of exons per gene | Average exon length (bp) | Average  intron length (bp) |
| --- | --- | --- | --- | --- | --- | --- | --- |
| *De novo* | AUGUSTUS | 22,542 | 54,517.1 | 1,455.5 | 8.9 | 163.0 | 6,691.4 |
| Homolog | Cat | 19,579 | 47,646.5 | 1,690.4 | 10.3 | 164.8 | 4,861.2 |
|  | Dog | 19,890 | 48,904.2 | 1,704.6 | 10.2 | 166.9 | 5,000.9 |
|  | Human | 20,196 | 56,969.7 | 1,732.8 | 10.3 | 167.7 | 5,755.1 |
|  | Mouse | 22,065 | 43,752.5 | 1,655.5 | 9.6 | 173.3 | 4,774.0 |
|  | Tiger | 18,311 | 46,718.7 | 1,671.3 | 10.5 | 159.5 | 4,669.4 |
| Final | | 19,043 | 34,265.9 | 1,618.4 | 9.4 | 171.6 | 3,947.6 |

**Table S8. Statistics regarding transposable elements (TEs) in leopard genome**

| Type | Ab initio based (bp) | Homology based (bp) | Total (bp) | Percentage of genome (%) |
| --- | --- | --- | --- | --- |
| DNA | 14,983,643 | 71,213,110 | 74,727,059 | 2.90 |
| LINE | 478,274,614 | 508,794,720 | 652,640,214 | 25.32 |
| LTR | 42,637,550 | 126,147,745 | 131,915,892 | 5.12 |
| Low_complexity | 6,186,939 | 6,515,731 | 7,272,828 | 0.28 |
| SINE | 4,687,033 | 71,002,632 | 72,009,528 | 2.79 |
| Satellite | 652,202 | 650,297 | 1,251,867 | 0.05 |
| Simple_repeat | 44,149,867 | 44,928,533 | 48,624,207 | 1.89 |
| TandemRepeat* |  |  | 67,553,344 | 2.62 |
| Unknown | 15,308,706 | 758,634 | 16,062,533 | 0.62 |
| Unspecified | 389,239 |  | 389,239 | 0.02 |
| Total | 605,050,886 | 829,976,231 | 1,006,545,511 | 39.04 |

* TandemRepeat was separately predicted using TRF program.

**Table S9. Assembly quality assessment using self-alignments**

| Library | | Number of   filtered reads | Number of   mapped reads | Percentage of  mapped reads |
| --- | --- | --- | --- | --- |
| 170bp | L1 | 649,639,158 | 648,380,590 | 99.81% |
|  | L2 | 645,441,596 | 644,116,197 | 99.79% |
| 400bp | L1 | 927,631,254 | 925,675,327 | 99.79% |
| 500bp | L1 | 355,755,802 | 355,021,715 | 99.79% |
| 700bp | L1 | 494,678,080 | 493,757,237 | 99.81% |
|  | L2 | 466,939,662 | 466,051,141 | 99.81% |
| 2kb | L1 | 141,024,484 | 140,465,545 | 99.60% |
|  | L2 | 157,681,268 | 157,027,375 | 99.59% |
|  | L3 | 165,113,480 | 164,509,242 | 99.63% |
| 5kb | L1 | 92,125,928 | 91,543,490 | 99.37% |
|  | L2 | 110,644,774 | 110,020,435 | 99.44% |
|  | L3 | 111,490,528 | 110,871,283 | 99.44% |
| 10kb | L1 | 88,451,252 | 87,990,350 | 99.48% |
|  | L2 | 71,257,114 | 70,805,035 | 99.37% |
|  | L3 | 76,850,626 | 76,376,319 | 99.38% |
| 15kb | L1 | 50,274,968 | 49,404,144 | 98.27% |
|  | L2 | 46,902,002 | 46,350,755 | 98.82% |
|  | L3 | 18,748,228 | 18,630,160 | 99.37% |
|  | L4 | 12,188,106 | 12,110,503 | 99.36% |
| 20kb | L1 | 47,273,942 | 46,217,382 | 97.77% |
|  | L2 | 48,418,062 | 47,879,499 | 98.89% |

**Table S10. Assembly results in five Felidae genomes**

|  | Leopard | Tiger | Cat | Cheetah | Lion |
| --- | --- | --- | --- | --- | --- |
| Assembly level | Scaffold | Scaffold | Chromosome | Scaffold | Scaffold |
| # sequences | 50,400 | 1,479 | 19 chromosomes + 267,606 unplaced scaffolds | 40,077 | 87,873 |
| Total bases (bp) | 2,578,022,254 | 2,391,082,183 | 2,641,342,258 | 2,375,874,546 | 2,442,522,584 |
| The longest length (bp) | 84,051,066 | 41,607,841 | 240,380,223 | 13,046,067 | 27,160,947 |
| The shortest length (bp) | 197 | 200 | 152 | 100 | 100 |
| Scaffold N50 (bp) | 21,701,857 | 8,860,407 | 142,431,058 | 3,121,442 | 4,005,654 |
| Contig N50 (bp) | 20,993 | 30,032 | 43,424 | 28,223 | 20,046 |
| GC contents | 41.71% | 41.40% | 41.92% | 41.30% | 41.27% |
| N bases | 3.85% | 2.44% | 1.58% | 1.77% | 3.32% |

**Table S11. Assembly and annotation quality assessment using single-copy orthologs mapping approach**

|  | Complete (%) | Duplicated (%) | Fragmented (%) | Missing (%) | Number of single-copy  orthologs genes |
| --- | --- | --- | --- | --- | --- |
| Leopard | 95 | 0.9 | 2.5 | 2.2 | 3,023 |
| Cat | 97 | 1.3 | 1.4 | 0.5 | 3,023 |
| Cheetah | 89 | 1.3 | 4.9 | 5.8 | 3,023 |
| Lion | 87 | 1.5 | 5.5 | 7.2 | 3,023 |
| Tiger | 93 | 0.6 | 4.3 | 2.0 | 3,023 |

**Table S12. Sequencing statistics regarding two wild Amur leopards and an Amur leopard cat**

| Sample | The number of raw read pairs | The number of proper  read pairs | The percentage of proper read pairs | Estimated  sequencing depth from raw read pairs | Estimated  sequencing depth  from proper read pairs |
| --- | --- | --- | --- | --- | --- |
| Amur leopard-01  (PPO1) | 463,914,011 | 383,291,526 | 82.62 | 38.66 | 31.94 |
| Amur leopard-02  (PPO5) | 457,450,100 | 382,230,035 | 83.56 | 38.12 | 31.85 |
| Amur leopard cat | 536,582,305 | 457,782,689 | 85.31 | 44.72 | 38.15 |

**Table S13. Mammalian genomes of three major dietary groups used in this study.** Gene numbers of leopard, lion, and cheetah were predicted, and other 15 mammalian genome information were from NCBI database.

| **Scientific name** | **Common name** | **Scaf. N50** | **# of genes** | **Assembly version** | **Order** | **Family** | **Genus** | **Diet** |
| --- | --- | --- | --- | --- | --- | --- | --- | --- |
| *Ailuropoda*  *Melanoleuca* | giant panda | 1.28 Mb | 19,589 | AilMel_1.0 | Carnivora | Ursidae | Ailuropoda | Herbivore |
| *Bos taurus* | cattle | 6.38 Mb | 21,618 | Bos_taurus_UMD_3.1.1 | Artiodactyla | Bovidae | Bos | Herbivore |
| *Equus caballus* | horse | 46.75 Mb | 19,874 | EquCab2.0 | Perissodactyla | Equidae | Equus | Herbivore |
| *Loxodonta*  *africana* | African savanna elephant | 46.40 Mb | 21,003 | Loxafr3.0 | Proboscidea | Elephantidae | Loxodonta | Herbivore |
| *Oryctolagus*  *Cuniculus* | rabbit | 35.97 Mb | 20,879 | OryCun2.0 | Lagomorpha | Leporidae | Oryctolagus | Herbivore |
| *Canis lupus*  *Familiaris* | domestic dog | 45.87 Mb | 20,009 | CanFam3.1 | Carnivora | Canidae | Canis | Omnivore |
| *Homo sapiens* | human | 59.36 Mb | 20,788 | GRCh38.p2 | Primates | Hominidae | Homo | Omnivore |
| *Monodelphis*  *domestica* | gray short-tailed opossum | 527 Mb | 21,017 | MonDom5 | Didelphimorphia | Didelphidae | Monodelphis | Omnivore |
| *Mus musculus* | house mouse | 52.59 Mb | 22,738 | GRCm38.p3 | Rodentia | Muridae | Mus | Omnivore |
| *Sus scrofa* | pig | 576 Kb | 24,233 | Sscrofa10.2 | Artiodactyla | Suidae | Sus | Omnivore |
| *Orcinus orca* | killer whale | 12.74 Mb | 18,200 | Oorc_1.1 | Cetartiodactyla | Delphinidae | Orcinus | Carnivore |
| *Felis catus* | domestic cat | 18.07 Mb | 19,713 | Felis_catus_8.0 | Carnivora | Felidae | Felis | Carnivore |
| *Panthera tigris* | tiger | 8.86 Mb | 18,385 | PanTig1.0 | Carnivora | Felidae | Panthera | Carnivore |
| *Panthera pardus* | leopard | 21.70 Mb | 19,043 | - | Carnivora | Felidae | Panthera | Carnivore |
| *Panthera leo* | lion | 4.01 Mb | 16,199 | - | Carnivora | Felidae | Panthera | Carnivore |
| *Acinonyx jubatus* | cheetah | 3.12 Mb | 16,053 | - | Carnivora | Felidae | Acinonyx | Carnivore |
| *Ursus maritimus* | polar bear | 15.94 Mb | 19,570 | UrsMar_1.0 | Carnivora | Ursidae | Ursus | Carnivore |
| *Sarcophilus*  *harrisii* | Tasmanian devil | 1.85 Mb | 19,562 | Devil_ref v7.0 | Dasyuromorphia | Dasyuridae | Sarcophilus | Carnivore |

**Table S14. GO enrichment of genes that were expanded in Felidae compared to Carnivora order.** Only GO categories with *P* < 0.0001 are shown.

| Term | Count | *P*-value | FDR |
| --- | --- | --- | --- |
| GO:0032982~myosin filament | 11 | 2.29E-18 | 2.62E-15 |
| GO:0016459~myosin complex | 14 | 4.16E-17 | 4.76E-14 |
| GO:0007608~sensory perception of smell | 29 | 8.83E-17 | 1.55E-13 |
| GO:0004984~olfactory receptor activity | 29 | 7.15E-16 | 6.88E-13 |
| GO:0007606~sensory perception of chemical stimulus | 29 | 1.36E-15 | 1.88E-12 |
| GO:0006350~transcription | 57 | 1.62E-15 | 2.34E-12 |
| GO:0006355~regulation of transcription, DNA-dependent | 52 | 2.87E-15 | 4.05E-12 |
| GO:0051252~regulation of RNA metabolic process | 52 | 7.19E-15 | 1.01E-11 |
| GO:0005859~muscle myosin complex | 9 | 1.14E-13 | 1.30E-10 |
| GO:0008270~zinc ion binding | 59 | 2.11E-13 | 2.20E-10 |
| GO:0016460~myosin II complex | 9 | 2.87E-13 | 3.28E-10 |
| GO:0045449~regulation of transcription | 59 | 1.14E-12 | 1.60E-09 |
| GO:0003677~DNA binding | 58 | 1.22E-12 | 1.27E-09 |
| GO:0000146~microfilament motor activity | 9 | 1.42E-12 | 1.48E-09 |
| GO:0007186~G-protein coupled receptor protein signaling pathway | 35 | 1.54E-10 | 2.16E-07 |
| GO:0007600~sensory perception | 29 | 4.96E-10 | 6.97E-07 |
| GO:0046914~transition metal ion binding | 59 | 5.88E-10 | 6.12E-07 |
| GO:0030048~actin filament-based movement | 8 | 8.87E-10 | 1.25E-06 |
| GO:0003774~motor activity | 14 | 1.09E-09 | 1.14E-06 |
| GO:0015629~actin cytoskeleton | 14 | 4.71E-09 | 5.39E-06 |
| GO:0050890~cognition | 29 | 6.53E-09 | 9.17E-06 |
| GO:0005516~calmodulin binding | 13 | 1.05E-08 | 1.09E-05 |
| GO:0005886~plasma membrane | 47 | 1.05E-08 | 1.21E-05 |
| GO:0043292~contractile fiber | 10 | 3.36E-08 | 3.85E-05 |
| GO:0050877~neurological system process | 31 | 2.26E-07 | 3.18E-04 |
| GO:0030016~myofibril | 9 | 2.61E-07 | 2.99E-04 |
| GO:0044449~contractile fiber part | 9 | 3.00E-07 | 3.43E-04 |
| GO:0044430~cytoskeletal part | 21 | 3.54E-07 | 4.05E-04 |
| GO:0030705~cytoskeleton-dependent intracellular transport | 8 | 3.90E-07 | 5.48E-04 |
| GO:0030898~actin-dependent ATPase activity | 5 | 5.75E-07 | 5.98E-04 |
| GO:0046872~metal ion binding | 66 | 3.21E-06 | 0.003343 |
| GO:0043169~cation binding | 66 | 4.57E-06 | 0.004761 |
| GO:0043167~ion binding | 66 | 7.91E-06 | 0.008236 |
| GO:0005863~striated muscle thick filament | 4 | 1.06E-05 | 0.012175 |
| GO:0007166~cell surface receptor linked signal transduction | 36 | 1.12E-05 | 0.015681 |
| GO:0003779~actin binding | 14 | 1.66E-05 | 0.01724 |
| GO:0005856~cytoskeleton | 22 | 2.93E-05 | 0.033583 |
| GO:0008307~structural constituent of muscle | 6 | 5.07E-05 | 0.052786 |
| GO:0030049~muscle filament sliding | 4 | 5.78E-05 | 0.081 |
| GO:0033275~actin-myosin filament sliding | 4 | 5.78E-05 | 0.081 |
| GO:0070252~actin-mediated cell contraction | 4 | 5.78E-05 | 0.081 |
| GO:0006936~muscle contraction | 9 | 7.22E-05 | 0.109 |

**Table S15. KEGG pathway enrichment of genes that were expanded in Felidae compared to Carnivora order**

| Term | Count | *P-*value | FDR |
| --- | --- | --- | --- |
| hsa04740:Olfactory transduction | 28 | 7.26E-19 | 5.05E-16 |
| hsa05416:Viral myocarditis | 14 | 3.20E-14 | 2.23E-11 |
| hsa04530:Tight junction | 15 | 8.85E-12 | 6.15E-09 |
| hsa05322:Systemic lupus erythematosus | 6 | 0.0022 | 1.54 |

**Table S16. GO enrichment of genes that were contracted in Felidae compared to Carnivora order.** Only GO categories with *P* < 1.0E-08 are shown.

| Term | Count | *P*-value | FDR |
| --- | --- | --- | --- |
| GO:0007156~homophilic cell adhesion | 61 | 3.21E-49 | 5.30E-46 |
| GO:0045095~keratin filament | 39 | 3.68E-28 | 4.88E-25 |
| GO:0016337~cell-cell adhesion | 61 | 4.48E-28 | 7.41E-25 |
| GO:0007606~sensory perception of chemical stimulus | 78 | 8.93E-27 | 1.48E-23 |
| GO:0007608~sensory perception of smell | 70 | 7.18E-24 | 1.19E-20 |
| GO:0004984~olfactory receptor activity | 69 | 4.69E-22 | 6.71E-19 |
| GO:0005882~intermediate filament | 45 | 1.87E-20 | 2.48E-17 |
| GO:0045111~intermediate filament cytoskeleton | 45 | 4.64E-20 | 6.14E-17 |
| GO:0007018~microtubule-based movement | 32 | 4.05E-18 | 6.69E-15 |
| GO:0007600~sensory perception | 88 | 4.98E-18 | 8.24E-15 |
| GO:0005328~neurotransmitter:sodium symporter activity | 16 | 2.07E-17 | 2.97E-14 |
| GO:0050877~neurological system process | 110 | 7.87E-17 | 1.89E-13 |
| GO:0005509~calcium ion binding | 95 | 9.52E-17 | 1.55E-13 |
| GO:0005886~plasma membrane | 262 | 6.28E-16 | 8.77E-13 |
| GO:0005326~neurotransmitter transporter activity | 16 | 1.48E-15 | 2.07E-12 |
| GO:0007155~cell adhesion | 76 | 2.14E-15 | 3.49E-12 |
| GO:0022610~biological adhesion | 76 | 2.25E-15 | 3.67E-12 |
| GO:0050890~cognition | 89 | 2.75E-15 | 4.59E-12 |
| GO:0016339~calcium-dependent cell-cell adhesion | 15 | 1.24E-14 | 2.04E-11 |
| GO:0016712~oxidoreductase activity, acting on paired  donors, with incorporation or reduction of molecular  oxygen, reduced flavin or flavoprotein as one donor, and  incorporation of one atom of oxygen | 16 | 1.43E-13 | 2.04E-10 |
| GO:0016021~integral to membrane | 327 | 1.68E-13 | 2.23E-10 |
| GO:0031224~intrinsic to membrane | 331 | 3.03E-12 | 4.01E-09 |
| GO:0070330~aromatase activity | 14 | 3.26E-12 | 4.66E-09 |
| GO:0020037~heme binding | 26 | 2.26E-11 | 3.23E-08 |
| GO:0005506~iron ion binding | 42 | 3.72E-11 | 5.33E-08 |
| GO:0030286~dynein complex | 15 | 8.07E-11 | 1.07E-07 |
| GO:0046906~tetrapyrrole binding | 26 | 9.78E-11 | 1.40E-07 |
| GO:0016887~ATPase activity | 43 | 1.33E-10 | 1.91E-07 |
| GO:0044430~cytoskeletal part | 88 | 1.55E-10 | 2.05E-07 |
| GO:0007186~G-protein coupled receptor protein signaling  pathway | 89 | 3.64E-10 | 6.02E-07 |
| GO:0042626~ATPase activity, coupled to transmembrane  movement of substances | 23 | 5.83E-10 | 8.35E-07 |
| GO:0043492~ATPase activity, coupled to movement of  substances | 23 | 7.01E-10 | 1.00E-06 |
| GO:0016820~hydrolase activity, acting on acid anhydrides,  catalyzing transmembrane movement of substances | 23 | 8.41E-10 | 1.20E-06 |
| GO:0004012~phospholipid-translocating ATPase activity | 10 | 1.42E-09 | 2.03E-06 |
| GO:0015247~aminophospholipid transporter activity | 10 | 1.42E-09 | 2.03E-06 |
| GO:0015370~solute:sodium symporter activity | 16 | 1.76E-09 | 2.52E-06 |
| GO:0051258~protein polymerization | 15 | 3.91E-09 | 6.47E-06 |
| GO:0015399~primary active transmembrane transporter  activity | 23 | 5.43E-09 | 7.77E-06 |
| GO:0015405~P-P-bond-hydrolysis-driven transmembrane  transporter activity | 23 | 5.43E-09 | 7.77E-06 |
| GO:0005858~axonemal dynein complex | 9 | 7.22E-09 | 9.56E-06 |

**Table S17. KEGG pathway enrichment of genes that were contracted in Felidae compared to Carnivora order**

| Term | Count | *P*-value | FDR |
| --- | --- | --- | --- |
| hsa00982:Drug metabolism | 29 | 6.83E-21 | 7.81E-18 |
| hsa04740:Olfactory transduction | 64 | 7.99E-19 | 9.14E-16 |
| hsa00830:Retinol metabolism | 25 | 7.60E-18 | 8.70E-15 |
| hsa00980:Metabolism of xenobiotics by cytochrome P450 | 26 | 1.02E-17 | 1.17E-14 |
| hsa00983:Drug metabolism | 19 | 4.39E-13 | 5.03E-10 |
| hsa04612:Antigen processing and presentation | 24 | 5.92E-12 | 6.78E-09 |
| hsa02010:ABC transporters | 18 | 9.63E-12 | 1.10E-08 |
| hsa05320:Autoimmune thyroid disease | 19 | 1.40E-11 | 1.60E-08 |
| hsa00053:Ascorbate and aldarate metabolism | 11 | 1.21E-09 | 1.38E-06 |
| hsa05130:Pathogenic Escherichia coli infection | 17 | 9.12E-09 | 1.04E-05 |
| hsa00040:Pentose and glucuronate interconversions | 10 | 5.70E-08 | 6.53E-05 |
| hsa04140:Regulation of autophagy | 13 | 5.82E-08 | 6.66E-05 |
| hsa00140:Steroid hormone biosynthesis | 14 | 2.16E-07 | 2.47E-04 |
| hsa00500:Starch and sucrose metabolism | 13 | 5.62E-07 | 6.43E-04 |
| hsa00591:Linoleic acid metabolism | 10 | 5.20E-06 | 0.0059 |
| hsa04622:RIG-I-like receptor signaling pathway | 15 | 8.19E-06 | 0.0093 |
| hsa04623:Cytosolic DNA-sensing pathway | 13 | 1.24E-05 | 0.014 |
| hsa00590:Arachidonic acid metabolism | 13 | 1.51E-05 | 0.017 |
| hsa00860:Porphyrin and chlorophyll metabolism | 10 | 2.32E-05 | 0.026 |
| hsa00150:Androgen and estrogen metabolism | 10 | 6.28E-05 | 0.071 |
| hsa04540:Gap junction | 15 | 1.15E-04 | 0.13 |
| hsa04650:Natural killer cell mediated cytotoxicity | 18 | 3.04E-04 | 0.34 |
| hsa04620:Toll-like receptor signaling pathway | 15 | 4.51E-04 | 0.51 |
| hsa05322:Systemic lupus erythematosus | 14 | 0.0012 | 1.37 |
| hsa00232:Caffeine metabolism | 4 | 0.0037 | 4.21 |

**Table S18. GO enrichment of genes that were expanded in Carnivora order compared to the common ancestor of carnivorans and horse**

| Term | Count | *P*-value | FDR |
| --- | --- | --- | --- |
| GO:0006355~regulation of transcription, DNA-dependent | 60 | 9.76E-26 | 1.28E-22 |
| GO:0051252~regulation of RNA metabolic process | 60 | 3.18E-25 | 4.16E-22 |
| GO:0045095~keratin filament | 18 | 6.13E-25 | 6.01E-22 |
| GO:0006350~transcription | 60 | 6.91E-22 | 9.04E-19 |
| GO:0008270~zinc ion binding | 66 | 1.03E-21 | 1.09E-18 |
| GO:0005882~intermediate filament | 18 | 2.91E-19 | 2.85E-16 |
| GO:0045111~intermediate filament cytoskeleton | 18 | 4.22E-19 | 4.14E-16 |
| GO:0045449~regulation of transcription | 62 | 9.38E-19 | 1.23E-15 |
| GO:0003677~DNA binding | 62 | 2.15E-18 | 2.27E-15 |
| GO:0046914~transition metal ion binding | 67 | 4.99E-18 | 5.27E-15 |
| GO:0046872~metal ion binding | 71 | 2.33E-11 | 2.45E-08 |
| GO:0043169~cation binding | 71 | 3.76E-11 | 3.97E-08 |
| GO:0043167~ion binding | 71 | 7.97E-11 | 8.40E-08 |
| GO:0044430~cytoskeletal part | 18 | 1.05E-07 | 1.03E-04 |
| GO:0005856~cytoskeleton | 18 | 1.85E-05 | 0.018 |
| GO:0007608~sensory perception of smell | 14 | 2.83E-05 | 0.037 |
| GO:0007606~sensory perception of chemical stimulus | 14 | 8.18E-05 | 0.107 |
| GO:0004984~olfactory receptor activity | 14 | 8.50E-05 | 0.089 |
| GO:0005198~structural molecule activity | 17 | 1.04E-04 | 0.10 |
| GO:0008305~integrin complex | 4 | 2.15E-04 | 0.21 |
| GO:0007229~integrin-mediated signaling pathway | 5 | 0.0021 | 2.74 |
| GO:0005200~structural constituent of cytoskeleton | 5 | 0.0037 | 3.93 |
| GO:0007600~sensory perception | 15 | 0.0038 | 4.97 |
| GO:0050890~cognition | 16 | 0.0043 | 5.50 |
| GO:0043232~intracellular non-membrane-bounded organelle | 20 | 0.0049 | 4.70 |
| GO:0043228~non-membrane-bounded organelle | 20 | 0.0049 | 4.70 |
| GO:0005886~plasma membrane | 25 | 0.0081 | 7.74 |

**Table S19. KEGG pathway enrichment of genes that were expanded in Carnivora order compared to the common ancestor of carnivorans and horse**

| Term | Count | *P*-value | FDR |
| --- | --- | --- | --- |
| hsa04740:Olfactory transduction | 13 | 3.06E-10 | 1.76E-07 |

**Table S20. GO enrichment of genes that were contracted in Carnivora order compared to the common ancestor of carnivorans and horse.** Only GO categories with *P* < 1.0E-07 are shown.

| Term | Count | *P*-value | FDR |
| --- | --- | --- | --- |
| GO:0007156~homophilic cell adhesion | 47 | 2.20E-42 | 3.22E-39 |
| GO:0016337~cell-cell adhesion | 47 | 1.38E-26 | 2.02E-23 |
| GO:0016712~oxidoreductase activity, acting on paired donors,  with incorporation or reduction of molecular oxygen,  reduced flavin or flavoprotein as one donor, and  incorporation of one atom of oxygen | 21 | 1.97E-26 | 2.44E-23 |
| GO:0070330~aromatase activity | 19 | 7.27E-25 | 9.01E-22 |
| GO:0005792~microsome | 39 | 7.72E-24 | 8.85E-21 |
| GO:0042598~vesicular fraction | 39 | 2.27E-23 | 2.60E-20 |
| GO:0046872~metal ion binding | 176 | 1.99E-22 | 2.47E-19 |
| GO:0043169~cation binding | 176 | 6.18E-22 | 7.66E-19 |
| GO:0043167~ion binding | 176 | 3.60E-21 | 4.47E-18 |
| GO:0045095~keratin filament | 24 | 8.35E-20 | 9.57E-17 |
| GO:0020037~heme binding | 27 | 1.90E-18 | 2.35E-15 |
| GO:0007608~sensory perception of smell | 47 | 2.02E-18 | 2.96E-15 |
| GO:0046906~tetrapyrrole binding | 27 | 1.03E-17 | 1.27E-14 |
| GO:0007606~sensory perception of chemical stimulus | 47 | 1.15E-16 | 1.67E-13 |
| GO:0004984~olfactory receptor activity | 44 | 1.83E-16 | 2.78E-13 |
| GO:0005886~plasma membrane | 137 | 1.53E-15 | 1.78E-12 |
| GO:0019825~oxygen binding | 16 | 1.47E-14 | 1.83E-11 |
| GO:0016339~calcium-dependent cell-cell adhesion | 13 | 2.14E-14 | 3.13E-11 |
| GO:0046914~transition metal ion binding | 122 | 2.43E-14 | 3.01E-11 |
| GO:0009055~electron carrier activity | 29 | 1.52E-13 | 1.89E-10 |
| GO:0031224~intrinsic to membrane | 167 | 7.99E-13 | 9.16E-10 |
| GO:0005882~intermediate filament | 24 | 1.86E-12 | 2.13E-09 |
| GO:0045111~intermediate filament cytoskeleton | 24 | 2.95E-12 | 3.38E-09 |
| GO:0016021~integral to membrane | 160 | 1.41E-11 | 1.61E-08 |
| GO:0007155~cell adhesion | 48 | 4.01E-11 | 5.86E-08 |
| GO:0022610~biological adhesion | 48 | 4.14E-11 | 6.05E-08 |
| GO:0007600~sensory perception | 50 | 5.48E-10 | 8.00E-07 |
| GO:0015020~glucuronosyltransferase activity | 10 | 5.83E-10 | 7.22E-07 |
| GO:0005624~membrane fraction | 44 | 3.30E-09 | 3.78E-06 |
| GO:0008270~zinc ion binding | 95 | 3.42E-09 | 4.25E-06 |
| GO:0005509~calcium ion binding | 51 | 7.15E-09 | 8.86E-06 |
| GO:0005506~iron ion binding | 27 | 9.64E-09 | 1.19E-05 |
| GO:0005626~insoluble fraction | 44 | 9.81E-09 | 1.12E-05 |
| GO:0050890~cognition | 50 | 2.41E-08 | 3.52E-05 |
| GO:0050877~neurological system process | 60 | 2.82E-08 | 4.12E-05 |
| GO:0007416~synaptogenesis | 10 | 3.06E-08 | 4.46E-05 |
| GO:0007186~G-protein coupled receptor protein signaling pathway | 57 | 3.18E-08 | 4.64E-05 |
| GO:0005783~endoplasmic reticulum | 46 | 6.19E-08 | 7.09E-05 |

**Table S21. KEGG pathway enrichment of genes that were contracted in Carnivora order compared to the common ancestor of carnivorans and horse**

| Term | Count | *P*-value | FDR |
| --- | --- | --- | --- |
| hsa00830:Retinol metabolism | 25 | 1.81E-25 | 1.80E-22 |
| hsa00980:Metabolism of xenobiotics by cytochrome P450 | 25 | 4.11E-24 | 4.09E-21 |
| hsa00982:Drug metabolism | 24 | 3.15E-22 | 3.14E-19 |
| hsa00983:Drug metabolism | 19 | 1.09E-18 | 1.09E-15 |
| hsa04740:Olfactory transduction | 43 | 5.06E-18 | 5.04E-15 |
| hsa00140:Steroid hormone biosynthesis | 18 | 1.34E-16 | 1.11E-13 |
| hsa00053:Ascorbate and aldarate metabolism | 10 | 5.06E-11 | 5.04E-08 |
| hsa00040:Pentose and glucuronate interconversions | 10 | 9.91E-11 | 9.86E-08 |
| hsa00590:Arachidonic acid metabolism | 14 | 4.93E-10 | 4.91E-07 |
| hsa00591:Linoleic acid metabolism | 11 | 5.07E-10 | 5.04E-07 |
| hsa00150:Androgen and estrogen metabolism | 11 | 1.12E-08 | 1.11E-05 |
| hsa00860:Porphyrin and chlorophyll metabolism | 10 | 5.76E-08 | 5.73E-05 |
| hsa00500:Starch and sucrose metabolism | 10 | 5.52E-07 | 5.50E-04 |
| hsa04612:Antigen processing and presentation | 9 | 8.97E-04 | 0.88 |
| hsa04650:Natural killer cell mediated cytotoxicity | 11 | 0.0014 | 1.45 |
| hsa00790:Folate biosynthesis | 4 | 0.0020 | 2.04 |
| hsa05130:Pathogenic Escherichia coli infection | 7 | 0.0025 | 2.50 |

**Table S22. GO enrichment of genes that were expanded in killer whale compared to the common ancestor of killer whale and cow**

| Term | Count | *P*-value | FDR |
| --- | --- | --- | --- |
| GO:0007156~homophilic cell adhesion | 17 | 2.51E-20 | 3.57E-17 |
| GO:0016337~cell-cell adhesion | 18 | 1.81E-16 | 3.11E-13 |
| GO:0043169~cation binding | 44 | 9.14E-13 | 1.02E-09 |
| GO:0043167~ion binding | 44 | 1.60E-12 | 1.79E-09 |
| GO:0046872~metal ion binding | 43 | 5.01E-12 | 5.59E-09 |
| GO:0007155~cell adhesion | 18 | 6.32E-10 | 8.98E-07 |
| GO:0022610~biological adhesion | 18 | 6.46E-10 | 9.18E-07 |
| GO:0005509~calcium ion binding | 19 | 1.43E-08 | 1.60E-05 |
| GO:0005886~plasma membrane | 23 | 2.28E-05 | 0.025 |
| GO:0005242~inward rectifier potassium channel activity | 4 | 9.90E-05 | 0.11 |
| GO:0006350~transcription | 20 | 2.29E-04 | 0.32 |
| GO:0031224~intrinsic to membrane | 25 | 0.0010 | 1.12 |
| GO:0008270~zinc ion binding | 20 | 0.0016 | 1.87 |
| GO:0016021~integral to membrane | 24 | 0.0018 | 1.99 |
| GO:0006355~regulation of transcription, DNA-dependent | 16 | 0.0025 | 3.62 |
| GO:0006986~response to unfolded protein | 4 | 0.0026 | 3.75 |
| GO:0051252~regulation of RNA metabolic process | 16 | 0.0032 | 4.49 |
| GO:0045449~regulation of transcription | 20 | 0.0033 | 4.62 |
| GO:0016339~calcium-dependent cell-cell adhesion | 3 | 0.0036 | 5.01 |
| GO:0005249~voltage-gated potassium channel activity | 4 | 0.0082 | 8.85 |

**Table S23. KEGG pathway enrichment of genes that were expanded in killer whale compared to the common ancestor of killer whale and cow**

| Term | Count | *P*-value | FDR |
| --- | --- | --- | --- |
| hsa04612:Antigen processing and presentation | 5 | 7.82E-06 | 0.0046 |
| hsa03040:Spliceosome | 4 | 0.0011 | 0.66 |
| hsa04144:Endocytosis | 4 | 0.0033 | 1.98 |
| hsa04010:MAPK signaling pathway | 4 | 0.0094 | 5.56 |

**Table S24. GO enrichment of genes that were contracted in killer whale compared to the common ancestor of killer whale and cow.** Only GO categories with *P* < 1.0E-07 are shown.

| Term | Count | *P*-value | FDR |
| --- | --- | --- | --- |
| GO:0007608~sensory perception of smell | 280 | 9.97E-237 | 1.66E-233 |
| GO:0007606~sensory perception of chemical stimulus | 291 | 1.12E-234 | 1.87E-231 |
| GO:0004984~olfactory receptor activity | 278 | 7.88E-230 | 1.13E-226 |
| GO:0007600~sensory perception | 300 | 7.93E-161 | 1.32E-157 |
| GO:0050890~cognition | 300 | 2.07E-144 | 3.46E-141 |
| GO:0007186~G-protein coupled receptor protein signaling pathway | 322 | 3.79E-136 | 6.33E-133 |
| GO:0050877~neurological system process | 302 | 1.84E-108 | 3.07E-105 |
| GO:0007166~cell surface receptor linked signal transduction | 343 | 2.23E-85 | 3.73E-82 |
| GO:0005886~plasma membrane | 438 | 2.39E-49 | 3.15E-46 |
| GO:0045095~keratin filament | 54 | 5.40E-41 | 7.11E-38 |
| GO:0016021~integral to membrane | 524 | 5.93E-41 | 7.81E-38 |
| GO:0031224~intrinsic to membrane | 532 | 3.30E-39 | 4.35E-36 |
| GO:0005882~intermediate filament | 65 | 4.51E-31 | 5.94E-28 |
| GO:0045111~intermediate filament cytoskeleton | 65 | 1.91E-30 | 2.52E-27 |
| GO:0016712~oxidoreductase activity, acting on paired donors,  with incorporation or reduction of molecular oxygen,  reduced flavin or flavoprotein as one donor,  and incorporation of one atom of oxygen | 21 | 3.53E-17 | 5.05E-14 |
| GO:0070330~aromatase activity | 19 | 1.63E-16 | 1.55E-13 |
| GO:0020037~heme binding | 36 | 4.16E-14 | 5.95E-11 |
| GO:0046906~tetrapyrrole binding | 36 | 3.41E-13 | 4.87E-10 |
| GO:0042626~ATPase activity, coupled to transmembrane movement of substances | 29 | 3.22E-10 | 4.60E-07 |
| GO:0043492~ATPase activity, coupled to movement of substances | 29 | 4.05E-10 | 5.79E-07 |
| GO:0016820~hydrolase activity, acting on acid anhydrides,  catalyzing transmembrane movement of substances | 29 | 5.08E-10 | 7.26E-07 |
| GO:0006952~defense response | 80 | 2.01E-09 | 3.36E-06 |
| GO:0015405~P-P-bond-hydrolysis-driven transmembrane transporter activity | 29 | 5.10E-09 | 7.29E-06 |
| GO:0015399~primary active transmembrane transporter activity | 29 | 5.10E-09 | 7.29E-06 |
| GO:0030286~dynein complex | 15 | 7.35E-09 | 9.67E-06 |
| GO:0042598~vesicular fraction | 41 | 3.33E-08 | 4.39E-05 |
| GO:0015020~glucuronosyltransferase activity | 12 | 3.76E-08 | 5.38E-05 |
| GO:0005792~microsome | 40 | 4.45E-08 | 5.86E-05 |
| GO:0016887~ATPase activity | 51 | 4.75E-08 | 6.79E-05 |
| GO:0019825~oxygen binding | 16 | 5.05E-08 | 7.22E-05 |
| GO:0042612~MHC class I protein complex | 13 | 5.17E-08 | 6.81E-05 |
| GO:0033559~unsaturated fatty acid metabolic process | 17 | 6.35E-08 | 1.06E-04 |
| GO:0004012~phospholipid-translocating ATPase activity | 10 | 8.46E-08 | 1.21E-04 |
| GO:0015247~aminophospholipid transporter activity | 10 | 8.46E-08 | 1.21E-04 |

**Table S25. KEGG pathway enrichment of genes that were contracted in killer whale compared to the common ancestor of killer whale and cow**

| Term | Count | *P*-value | FDR |
| --- | --- | --- | --- |
| hsa04740:Olfactory transduction | 266 | 1.40E-208 | 1.59E-205 |
| hsa02010:ABC transporters | 27 | 3.96E-16 | 5.00E-13 |
| hsa00980:Metabolism of xenobiotics by cytochrome P450 | 30 | 1.09E-14 | 1.23E-11 |
| hsa00982:Drug metabolism | 30 | 3.25E-14 | 3.69E-11 |
| hsa00830:Retinol metabolism | 26 | 2.71E-12 | 3.07E-09 |
| hsa04612:Antigen processing and presentation | 29 | 1.15E-09 | 1.30E-06 |
| hsa00983:Drug metabolism | 20 | 3.24E-09 | 3.67E-06 |
| hsa04650:Natural killer cell mediated cytotoxicity | 36 | 1.71E-08 | 1.94E-05 |
| hsa00590:Arachidonic acid metabolism | 21 | 9.74E-08 | 1.10E-04 |
| hsa00140:Steroid hormone biosynthesis | 18 | 5.15E-07 | 5.84E-04 |
| hsa00591:Linoleic acid metabolism | 13 | 4.13E-06 | 0.0046 |
| hsa00053:Ascorbate and aldarate metabolism | 10 | 8.14E-06 | 0.00922 |
| hsa05320:Autoimmune thyroid disease | 17 | 1.31E-05 | 0.014 |
| hsa00040:Pentose and glucuronate interconversions | 10 | 1.49E-05 | 0.016 |
| hsa04140:Regulation of autophagy | 13 | 6.02E-05 | 0.068 |
| hsa00500:Starch and sucrose metabolism | 13 | 4.25E-04 | 0.48 |
| hsa00150:Androgen and estrogen metabolism | 11 | 0.0020 | 2.27 |
| hsa00860:Porphyrin and chlorophyll metabolism | 10 | 0.0031 | 3.51 |
| hsa04623:Cytosolic DNA-sensing pathway | 13 | 0.0052 | 5.81 |

**Table S26. GO enrichment of genes that were expanded in Tasmanian devil compared to the common ancestor of Tasmanian devil and opossum.** Only GO categories with *P* < 1.0E-08 are shown.

| Term | Count | *P*-value | FDR |
| --- | --- | --- | --- |
| GO:0003774~motor activity | 22 | 2.53E-20 | 3.13E-17 |
| GO:0030286~dynein complex | 14 | 2.45E-18 | 3.04E-15 |
| GO:0005003~ephrin receptor activity | 11 | 3.35E-17 | 4.15E-14 |
| GO:0003777~microtubule motor activity | 16 | 1.20E-16 | 1.33E-13 |
| GO:0005524~ATP binding | 48 | 2.29E-16 | 2.78E-13 |
| GO:0032559~adenyl ribonucleotide binding | 48 | 4.98E-16 | 5.55E-13 |
| GO:0016887~ATPase activity | 25 | 1.07E-15 | 1.38E-12 |
| GO:0030554~adenyl nucleotide binding | 48 | 3.57E-15 | 4.40E-12 |
| GO:0001883~purine nucleoside binding | 48 | 6.40E-15 | 7.97E-12 |
| GO:0001882~nucleoside binding | 48 | 8.29E-15 | 1.03E-11 |
| GO:0007018~microtubule-based movement | 16 | 4.03E-14 | 6.06E-11 |
| GO:0005930~axoneme | 12 | 1.03E-13 | 1.28E-10 |
| GO:0044430~cytoskeletal part | 37 | 3.74E-13 | 4.64E-10 |
| GO:0032555~purine ribonucleotide binding | 48 | 1.12E-12 | 1.39E-09 |
| GO:0032553~ribonucleotide binding | 48 | 1.12E-12 | 1.39E-09 |
| GO:0031420~alkali metal ion binding | 19 | 1.16E-12 | 1.44E-09 |
| GO:0015301~anion:anion antiporter activity | 9 | 3.18E-12 | 3.94E-09 |
| GO:0005858~axonemal dynein complex | 8 | 4.90E-12 | 6.07E-09 |
| GO:0017076~purine nucleotide binding | 48 | 5.54E-12 | 6.86E-09 |
| GO:0001539~ciliary or flagellar motility | 8 | 9.86E-12 | 1.48E-08 |
| GO:0005875~microtubule associated complex | 14 | 1.45E-11 | 1.79E-08 |
| GO:0035085~cilium axoneme | 9 | 5.51E-11 | 6.83E-08 |
| GO:0044447~axoneme part | 8 | 1.16E-10 | 1.43E-07 |
| GO:0015672~monovalent inorganic cation transport | 19 | 2.41E-10 | 3.62E-07 |
| GO:0004714~transmembrane receptor protein tyrosine kinase activity | 11 | 2.98E-10 | 3.69E-07 |
| GO:0000166~nucleotide binding | 49 | 3.97E-10 | 4.91E-07 |
| GO:0022843~voltage-gated cation channel activity | 14 | 4.79E-10 | 5.93E-07 |
| GO:0007017~microtubule-based process | 17 | 5.21E-10 | 7.84E-07 |
| GO:0001518~voltage-gated sodium channel complex | 7 | 6.19E-10 | 7.67E-07 |
| GO:0006811~ion transport | 27 | 1.40E-09 | 2.11E-06 |
| GO:0006812~cation transport | 23 | 1.70E-09 | 2.55E-06 |
| GO:0005248~voltage-gated sodium channel activity | 7 | 1.88E-09 | 2.33E-06 |
| GO:0030001~metal ion transport | 21 | 2.74E-09 | 4.12E-06 |
| GO:0034706~sodium channel complex | 7 | 3.27E-09 | 4.06E-06 |
| GO:0005856~cytoskeleton | 38 | 3.74E-09 | 4.63E-06 |
| GO:0031402~sodium ion binding | 12 | 5.84E-09 | 7.23E-06 |
| GO:0044463~cell projection part | 16 | 6.75E-09 | 8.36E-06 |
| GO:0044441~cilium part | 9 | 7.75E-09 | 9.61E-06 |

**Table S27. KEGG pathway enrichment of genes that were expanded in Tasmanian devil compared to the common ancestor of Tasmanian devil and opossum**

| Term | Count | *P*-value | FDR |
| --- | --- | --- | --- |
| hsa04360:Axon guidance | 11 | 3.38E-07 | 2.85E-04 |
| hsa04530:Tight junction | 9 | 3.99E-05 | 0.033 |
| hsa02010:ABC transporters | 6 | 6.10E-05 | 0.051 |
| hsa05416:Viral myocarditis | 6 | 5.99E-04 | 0.50 |
| hsa04512:ECM-receptor interaction | 6 | 0.0012 | 1.08 |

**Table S28. GO enrichment of genes that were contracted in Tasmanian devil compared to the common ancestor of Tasmanian devil and opossum.** Only GO categories with *P* < 1.0E-06 are shown.

| Term | Count | *P*-value | FDR |
| --- | --- | --- | --- |
| GO:0004984~olfactory receptor activity | 87 | 4.72E-37 | 6.64E-34 |
| GO:0007608~sensory perception of smell | 87 | 8.12E-37 | 1.36E-33 |
| GO:0007606~sensory perception of chemical stimulus | 87 | 2.91E-33 | 4.90E-30 |
| GO:0006355~regulation of transcription, DNA-dependent | 167 | 8.41E-28 | 1.41E-24 |
| GO:0051252~regulation of RNA metabolic process | 168 | 3.64E-27 | 6.11E-24 |
| GO:0006350~transcription | 175 | 4.69E-23 | 7.88E-20 |
| GO:0008270~zinc ion binding | 184 | 1.29E-22 | 1.81E-19 |
| GO:0003677~DNA binding | 185 | 1.33E-22 | 1.87E-19 |
| GO:0046914~transition metal ion binding | 207 | 3.17E-22 | 4.45E-19 |
| GO:0007600~sensory perception | 96 | 4.52E-22 | 7.59E-19 |
| GO:0050890~cognition | 99 | 4.08E-20 | 6.86E-17 |
| GO:0045449~regulation of transcription | 190 | 1.05E-18 | 1.77E-15 |
| GO:0005886~plasma membrane | 210 | 1.33E-17 | 1.73E-14 |
| GO:0007186~G-protein coupled receptor protein signaling pathway | 107 | 1.70E-17 | 2.86E-14 |
| GO:0050877~neurological system process | 112 | 2.06E-17 | 3.45E-14 |
| GO:0045095~keratin filament | 24 | 8.51E-15 | 1.11E-11 |
| GO:0016021~integral to membrane | 254 | 1.15E-13 | 1.50E-10 |
| GO:0031224~intrinsic to membrane | 259 | 3.27E-13 | 4.26E-10 |
| GO:0046872~metal ion binding | 242 | 1.22E-12 | 1.71E-09 |
| GO:0005328~neurotransmitter:sodium symporter activity | 13 | 1.38E-12 | 1.94E-09 |
| GO:0043169~cation binding | 243 | 1.92E-12 | 2.69E-09 |
| GO:0007166~cell surface receptor linked signal transduction | 135 | 2.19E-12 | 3.67E-09 |
| GO:0043167~ion binding | 244 | 5.63E-12 | 7.92E-09 |
| GO:0005326~neurotransmitter transporter activity | 13 | 2.56E-11 | 3.60E-08 |
| GO:0016712~oxidoreductase activity, acting on paired donors, with incorporation or reduction of molecular oxygen, reduced flavin or flavoprotein as one donor, and incorporation of one atom of oxygen | 13 | 6.58E-10 | 9.25E-07 |
| GO:0006836~neurotransmitter transport | 19 | 3.64E-09 | 6.12E-06 |
| GO:0045111~intermediate filament cytoskeleton | 25 | 1.71E-08 | 2.23E-05 |
| GO:0070330~aromatase activity | 11 | 1.82E-08 | 2.56E-05 |
| GO:0005882~intermediate filament | 24 | 5.06E-08 | 6.60E-05 |
| GO:0004222~metalloendopeptidase activity | 19 | 1.38E-07 | 1.94E-04 |
| GO:0016165~lipoxygenase activity | 6 | 5.81E-07 | 8.17E-04 |
| GO:0015370~solute:sodium symporter activity | 13 | 7.02E-07 | 9.87E-04 |

**Table S29. KEGG pathway enrichment of genes that were contracted in Tasmanian devil compared to the common ancestor of Tasmanian devil and opossum**

| Term | Count | *P*-value | FDR |
| --- | --- | --- | --- |
| hsa04740:Olfactory transduction | 81 | 2.06E-40 | 2.35E-37 |
| hsa00982:Drug metabolism | 16 | 9.63E-09 | 1.10E-05 |
| hsa00980:Metabolism of xenobiotics by cytochrome P450 | 14 | 3.92E-07 | 4.47E-04 |
| hsa00591:Linoleic acid metabolism | 10 | 7.08E-07 | 8.08E-04 |
| hsa00830:Retinol metabolism | 13 | 8.33E-07 | 9.50E-04 |
| hsa00590:Arachidonic acid metabolism | 13 | 1.26E-06 | 0.0014 |
| hsa05322:Systemic lupus erythematosus | 13 | 4.68E-04 | 0.53 |
| hsa04612:Antigen processing and presentation | 11 | 0.0014 | 1.64 |
| hsa04330:Notch signaling pathway | 8 | 0.0022 | 2.50 |
| hsa00983:Drug metabolism | 7 | 0.0063 | 7.01 |
| hsa00790:Folate biosynthesis | 4 | 0.0079 | 8.68 |

**Table S30. Gene copy evolution of UGT and AMY families**

| Diet | Species | Number of genes in each family | | | | | |
| --- | --- | --- | --- | --- | --- | --- | --- |
|  |  | UGT1 | UGT2 | UGT3 | UGT8 | UGT  total | AMY  total |
| Carnivores | Cat | 1 | 2 | 4 | 1 | 8 | 1 |
|  | Cheetah | 1 | 2 | 4 | 1 | 8 | 1 |
|  | Tiger | 1 | 3 | 5 | 1 | 10 | 1 |
|  | Lion | 1 | 2 | 4 | 1 | 8 | 1 |
|  | Leopard | 1 | 2 | 4 | 1 | 8 | 1 |
|  | Polar bear | 1 | 3 | 3 | 1 | 8 | 2 |
|  | Killer whale | 1 | 2 | 1 | 1 | 5 | 1 |
|  | Tasmanian devil | 3 | 1 | 0 | 1 | 5 | 1 |
| Omnivores | Human | 9 | 10 | 2 | 1 | 22 | 5 |
|  | Dog | 5 | 8 | 3 | 1 | 17 | 5 |
|  | Pig | 4 | 13 | 1 | 1 | 19 | 6 |
|  | Mouse | 8 | 10 | 2 | 1 | 21 | 7 |
|  | Opossum | 4 | 1 | 0 | 1 | 6 | 1 |
| Herbivores | Rabbit | 3 | 24 | 1 | 1 | 29 | 4 |
|  | Cow | 3 | 14 | 2 | 1 | 20 | 2 |
|  | Elephant | 10 | 16 | 1 | 1 | 28 | 2 |
|  | Horse | 5 | 10 | 2 | 1 | 18 | 2 |
|  | Panda | 2 | 4 | 3 | 1 | 10 | 2 |

**Table S31. Pseudogenization of *GCKR* gene in 14 mammalian genomes**

| Species | NCBI accession for *GCKR* gene | Status of *GCKR* gene | # of premature stop codon | # of frame-shift mutation |
| --- | --- | --- | --- | --- |
| Cow | XP_010808614.1 | Pseudogenized | 4 | 6 |
| Dog | NP_001273995.1 | Intact | - | - |
| Elephant | XP_003411994.1 | Intact | - | - |
| Ferret | XP_012918398.1 | Pseudogenized | 3 | 1 |
| Horse | XP_001502228.2 | Intact | - | - |
| Human | NP_001477.2 | Intact | - | - |
| Killer whale | XP_004268277.1 | Pseudogenized | 4 | 1 |
| Mouse | XP_006503944.1 | Intact | - | - |
| Opossum | XP_003339748.1 | Pseudogenized | 2 | - |
| Panda | XP_002913853.1 | Intact | - | - |
| Pig | XP_013843384.1 | Intact | - | - |
| Polar bear | XP_008698520.1 | Intact | - | - |
| Rabbit | XP_002709846.1 | Intact | - | - |
| Tasmanian devil | XP_003767172.1 | Intact | - | - |

**Table S32. GO enrichment of positively selected genes in leopard**

| Term | Count | *P*-value | FDR |
| --- | --- | --- | --- |
| GO:0031981~nuclear lumen | 71 | 6.20E-06 | 0.01 |
| GO:0005654~nucleoplasm | 47 | 5.00E-05 | 0.07 |
| GO:0031974~membrane-enclosed lumen | 81 | 7.56E-05 | 0.10 |
| GO:0070013~intracellular organelle lumen | 78 | 9.49E-05 | 0.13 |
| GO:0043233~organelle lumen | 79 | 1.15E-04 | 0.16 |
| GO:0005525~GTP binding | 24 | 1.31E-04 | 0.19 |
| GO:0032561~guanyl ribonucleotide binding | 24 | 1.92E-04 | 0.28 |
| GO:0019001~guanyl nucleotide binding | 24 | 1.92E-04 | 0.28 |
| GO:0007264~small GTPase mediated signal transduction | 21 | 3.25E-04 | 0.56 |
| GO:0042127~regulation of cell proliferation | 39 | 5.72E-04 | 0.99 |
| GO:0009057~macromolecule catabolic process | 38 | 9.55E-04 | 1.64 |
| GO:0044265~cellular macromolecule catabolic process | 36 | 9.61E-04 | 1.65 |
| GO:0006259~DNA metabolic process | 27 | 0.0018 | 3.10 |
| GO:0000930~gamma-tubulin complex | 4 | 0.0032 | 4.33 |
| GO:0022613~ribonucleoprotein complex biogenesis | 13 | 0.0043 | 7.24 |
| GO:0008274~gamma-tubulin ring complex | 3 | 0.0047 | 6.28 |
| GO:0000931~gamma-tubulin large complex | 3 | 0.0047 | 6.28 |
| GO:0007049~cell cycle | 35 | 0.0053 | 8.74 |

**Table S33. GO enrichment of shared positively selected genes in Felidae**

| Term | Count | *P*-value | FDR |
| --- | --- | --- | --- |
| GO:0044421~extracellular region part | 25 | 6.10E-05 | 0.08 |
| GO:0009897~external side of plasma membrane | 10 | 7.85E-05 | 0.10 |
| GO:0005578~proteinaceous extracellular matrix | 13 | 1.45E-04 | 0.18 |
| GO:0031012~extracellular matrix | 13 | 2.91E-04 | 0.37 |
| GO:0005539~glycosaminoglycan binding | 8 | 4.04E-04 | 0.54 |
| GO:0001871~pattern binding | 8 | 7.14E-04 | 0.96 |
| GO:0030247~polysaccharide binding | 8 | 7.14E-04 | 0.96 |
| GO:0009986~cell surface | 12 | 0.0011 | 1.44 |
| GO:0008201~heparin binding | 6 | 0.0032 | 4.17 |
| GO:0043066~negative regulation of apoptosis | 11 | 0.0038 | 6.03 |
| GO:0008289~lipid binding | 12 | 0.0041 | 5.38 |
| GO:0043069~negative regulation of programmed cell death | 11 | 0.0041 | 6.63 |
| GO:0060548~negative regulation of cell death | 11 | 0.0042 | 6.76 |
| GO:0007346~regulation of mitotic cell cycle | 7 | 0.0050 | 7.92 |
| GO:0006955~immune response | 16 | 0.0052 | 8.22 |
| GO:0005768~endosome | 10 | 0.0062 | 7.58 |

**Table S34. GO enrichment of shared positively selected genes in carnivores**

| Term | Count | *P*-value | FDR |
| --- | --- | --- | --- |
| GO:0044421~extracellular region part | 21 | 1.05E-04 | 0.13 |
| GO:0005576~extracellular region | 32 | 3.06E-04 | 0.38 |
| GO:0005539~glycosaminoglycan binding | 6 | 0.0040 | 5.06 |
| GO:0030246~carbohydrate binding | 9 | 0.0050 | 6.30 |
| GO:0008045~motor axon guidance | 3 | 0.0050 | 7.73 |
| GO:0005578~proteinaceous extracellular matrix | 9 | 0.0051 | 6.16 |
| GO:0030247~polysaccharide binding | 6 | 0.0059 | 7.47 |
| GO:0001871~pattern binding | 6 | 0.0059 | 7.47 |
| GO:0031012~extracellular matrix | 9 | 0.0079 | 9.41 |
| GO:0007411~axon guidance | 5 | 0.0120 | 17.60 |
| GO:0060348~bone development | 5 | 0.0190 | 26.60 |

**Table S35. GO enrichment of shared positively selected genes in omnivores**

| Term | Count | *P*-value | FDR |
| --- | --- | --- | --- |
| GO:0005201~extracellular matrix structural constituent | 8 | 1.01E-05 | 0.01 |
| GO:0005576~extracellular region | 38 | 1.10E-04 | 0.14 |
| GO:0019861~flagellum | 5 | 0.0011 | 1.45 |
| GO:0030317~sperm motility | 4 | 0.0015 | 2.42 |
| GO:0007130~synaptonemal complex assembly | 3 | 0.0017 | 2.74 |
| GO:0070193~synaptonemal complex organization | 3 | 0.0017 | 2.74 |
| GO:0044421~extracellular region part | 20 | 0.0032 | 4.04 |
| GO:0005578~proteinaceous extracellular matrix | 10 | 0.0049 | 6.11 |
| GO:0001669~acrosomal vesicle | 4 | 0.0059 | 7.42 |
| GO:0009566~fertilization | 5 | 0.0061 | 9.39 |
| GO:0034097~response to cytokine stimulus | 5 | 0.0061 | 9.39 |
| GO:0007129~synapsis | 3 | 0.0062 | 9.48 |
| GO:0070192~chromosome organization involved in meiosis | 3 | 0.0062 | 9.48 |
| GO:0031012~extracellular matrix | 10 | 0.0078 | 9.68 |

**Table S36. KEGG pathway enrichment of shared positively selected genes in omnivores**

| Term | Count | *P*-value | FDR |
| --- | --- | --- | --- |
| hsa04060:Cytokine-cytokine receptor interaction | 8 | 0.0051 | 4.79 |

**Table S37. GO enrichment of shared positively selected genes in herbivores**

| Term | Count | *P*-value | FDR |
| --- | --- | --- | --- |
| GO:0044421~extracellular region part | 19 | 7.79E-05 | 0.096339 |
| GO:0005576~extracellular region | 28 | 3.14E-04 | 0.388126 |
| GO:0005615~extracellular space | 14 | 7.82E-04 | 0.963411 |
| GO:0005125~cytokine activity | 7 | 9.71E-04 | 1.242315 |
| GO:0009566~fertilization | 5 | 0.0015 | 2.28 |
| GO:0005201~extracellular matrix structural constituent | 5 | 0.0016 | 2.04 |
| GO:0001669~acrosomal vesicle | 4 | 0.0021 | 2.53 |
| GO:0032101~regulation of response to external stimulus | 6 | 0.0031 | 4.77 |
| GO:0007596~blood coagulation | 5 | 0.0037 | 5.71 |
| GO:0050817~coagulation | 5 | 0.0037 | 5.71 |
| GO:0002694~regulation of leukocyte activation | 6 | 0.0037 | 5.71 |
| GO:0051347~positive regulation of transferase activity | 7 | 0.0038 | 5.79 |
| GO:0002696~positive regulation of leukocyte activation | 5 | 0.0043 | 6.52 |
| GO:0007599~hemostasis | 5 | 0.0045 | 6.96 |
| GO:0050865~regulation of cell activation | 6 | 0.0046 | 7.09 |
| GO:0050867~positive regulation of cell activation | 5 | 0.0050 | 7.64 |
| GO:0051052~regulation of DNA metabolic process | 5 | 0.0055 | 8.36 |
| GO:0032844~regulation of homeostatic process | 5 | 0.0055 | 8.36 |
| GO:0007338~single fertilization | 4 | 0.0061 | 9.27 |
| GO:0009897~external side of plasma membrane | 6 | 0.0063 | 7.55 |
| GO:0002703~regulation of leukocyte mediated immunity | 4 | 0.0064 | 9.69 |

**Table S38. KEGG pathway enrichment of shared positively selected genes in herbivores**

| Term | Count | *P*-value | FDR |
| --- | --- | --- | --- |
| hsa04640:Hematopoietic cell lineage | 5 | 7.36E-04 | 0.64 |
| hsa04060:Cytokine-cytokine receptor interaction | 7 | 0.0014 | 1.18 |
| hsa04512:ECM-receptor interaction | 4 | 0.0077 | 6.47 |

**Table S39. Variants statistics regarding mapping of Felidae raw reads to the cat reference (Felis_catus_8.0)**

| Species | All variant sites | Total number  of SNV sites | Homozygous  SNV sites | Heterozygous  SNV sites | Indel sites |
| --- | --- | --- | --- | --- | --- |
| Leopard | 52,946,286 | 47,321,889 | 45,495,382 | 1,826,507 | 5,624,397 |
| Amur leopard-01 | 52,537,072 | 46,988,478 | 45,766,378 | 1,222,100 | 5,548,594 |
| Amur leopard-02 | 52,968,234 | 47,371,008 | 45,971,258 | 1,399,750 | 5,597,226 |
| Lion | 50,247,149 | 45,268,011 | 41,421,655 | 3,846,356 | 4,979,138 |
| Lion-01 | 52,897,073 | 47,273,169 | 45,338,579 | 1,934,590 | 5,623,904 |
| White lion | 51,618,649 | 46,195,513 | 44,564,736 | 1,630,777 | 5,423,136 |
| Bengal tiger | 51,491,685 | 45,979,066 | 43,568,091 | 2,410,975 | 5,512,619 |
| Amur tiger | 51,057,530 | 45,861,367 | 43,157,393 | 2,703,974 | 5,196,163 |
| White tiger | 48,897,698 | 43,668,070 | 41,418,085 | 2,249,985 | 5,229,628 |
| Snow leopard | 52,483,709 | 46,887,759 | 45,770,403 | 1,117,356 | 5,595,950 |
| Leopard cat  (SRP059496) | 38,553,587 | 34,466,940 | 28,841,192 | 5,625,748 | 4,086,647 |
| Amur leopard cat | 42,502,163 | 37,469,246 | 32,982,479 | 4,486,767 | 5,032,917 |
| Cheetah | 36,987,255 | 32,935,228 | 31,790,223 | 1,145,005 | 4,052,027 |
| Boris cat  (SRP039031) | 12,295,095 | 10,512,963 | 3,609,859 | 6,903,104 | 1,782,132 |

**Table S40. GO enrichment of Felidae-specific genes having function altering amino acid changes**

| Term | Count | *P*-value | FDR |
| --- | --- | --- | --- |
| GO:0000279~M phase | 63 | 1.96E-13 | 3.53E-10 |
| GO:0022403~cell cycle phase | 69 | 1.07E-11 | 1.93E-08 |
| GO:0022402~cell cycle process | 81 | 3.25E-10 | 5.85E-07 |
| GO:0007049~cell cycle | 100 | 8.83E-10 | 1.59E-06 |
| GO:0006259~DNA metabolic process | 72 | 5.03E-09 | 9.06E-06 |
| GO:0000087~M phase of mitotic cell cycle | 42 | 6.23E-09 | 1.12E-05 |
| GO:0000280~nuclear division | 41 | 1.16E-08 | 2.09E-05 |
| GO:0007067~mitosis | 41 | 1.16E-08 | 2.09E-05 |
| GO:0048285~organelle fission | 41 | 3.71E-08 | 6.68E-05 |
| GO:0051301~cell division | 47 | 1.27E-07 | 2.28E-04 |
| GO:0000793~condensed chromosome | 28 | 1.69E-07 | 2.44E-04 |
| GO:0006974~response to DNA damage stimulus | 53 | 7.39E-07 | 0.0013 |
| GO:0043228~non-membrane-bounded organelle | 237 | 1.33E-06 | 0.0019 |
| GO:0043232~intracellular non-membrane-bounded organelle | 237 | 1.33E-06 | 0.0019 |
| GO:0044427~chromosomal part | 53 | 2.49E-06 | 0.0036 |
| GO:0005694~chromosome | 60 | 2.69E-06 | 0.0039 |
| GO:0000278~mitotic cell cycle | 50 | 6.70E-06 | 0.012 |
| GO:0051327~M phase of meiotic cell cycle | 21 | 8.97E-06 | 0.016 |
| GO:0007126~meiosis | 21 | 8.97E-06 | 0.016 |
| GO:0004518~nuclease activity | 27 | 9.53E-06 | 0.015 |
| GO:0006281~DNA repair | 41 | 1.10E-05 | 0.020 |
| GO:0051321~meiotic cell cycle | 21 | 1.23E-05 | 0.022 |
| GO:0000776~kinetochore | 18 | 1.54E-05 | 0.022 |
| GO:0005814~centriole | 11 | 2.77E-05 | 0.040 |
| GO:0000777~condensed chromosome kinetochore | 15 | 2.98E-05 | 0.043 |
| GO:0005819~spindle | 25 | 6.77E-05 | 0.098 |
| GO:0000779~condensed chromosome, centromeric region | 15 | 1.35E-04 | 0.20 |
| GO:0015630~microtubule cytoskeleton | 62 | 1.45E-04 | 0.21 |
| GO:0033554~cellular response to stress | 63 | 1.61E-04 | 0.29 |
| GO:0004519~endonuclease activity | 18 | 2.16E-04 | 0.34 |
| GO:0006310~DNA recombination | 19 | 2.65E-04 | 0.48 |
| GO:0044450~microtubule organizing center part | 13 | 3.64E-04 | 0.52 |
| GO:0000723~telomere maintenance | 9 | 4.33E-04 | 0.78 |
| GO:0032200~telomere organization | 9 | 5.62E-04 | 1.01 |
| GO:0070193~synaptonemal complex organization | 5 | 6.54E-04 | 1.17 |
| GO:0007130~synaptonemal complex assembly | 5 | 6.54E-04 | 1.17 |
| GO:0004896~cytokine receptor activity | 12 | 6.80E-04 | 1.07 |
| GO:0005739~mitochondrion | 103 | 7.70E-04 | 1.11 |
| GO:0000775~chromosome, centromeric region | 20 | 8.43E-04 | 1.21 |

**Table S41. KEGG pathway enrichment of Felidae-specific genes having function altering amino acid changes**

| Term | Count | *P*-value | FDR |
| --- | --- | --- | --- |
| hsa03450:Non-homologous end-joining | 6 | 6.68E-04 | 0.81 |
| hsa04060:Cytokine-cytokine receptor interaction | 28 | 0.0040 | 4.71 |

**Table S42. Variants statistics regarding mapping of Hominidae and Bovidae raw reads to the human and cow references**

| Family | Species | All variant  sites | Total number  of SNV sites | Homozygous  SNV sites | Heterozygous  SNV sites | Indel sites |
| --- | --- | --- | --- | --- | --- | --- |
| Hominidae | Bonobo | 33,290,642 | 30,447,841 | 27,915,325 | 2,532,516 | 2,842,801 |
|  | Chimpanzee | 37,897,572 | 34,600,658 | 28,830,656 | 5,770,002 | 3,296,914 |
|  | Gorilla | 45,198,660 | 41,452,878 | 36,172,009 | 5,280,869 | 3,745,782 |
|  | Orangutan | 84,426,470 | 78,815,738 | 71,088,342 | 7,727,396 | 5,610,732 |
| Bovidae | Goat | 111,574,672 | 105,750,483 | 99,847,134 | 5,903,349 | 5,824,189 |
|  | Sheep | 113,960,484 | 108,178,988 | 99,478,910 | 8,700,078 | 5,781,496 |
|  | Water  Buffalo | 60,916,988 | 56,964,575 | 49,345,127 | 7,619,448 | 3,952,413 |
|  | Yak | 21,285,532 | 19,538,552 | 15,873,089 | 3,665,463 | 1,746,980 |

**Table S43. Statistics regarding highly conserved regions in Felidae, Hominidae, and Bovidae genomes**

| Family | Reference  genome size (excluding unplaced fragments) | The number of windows (>80% of sufficiently covered) | | Highly conserved windows (Adjusted *P*-value < 0.0001) | | |
| --- | --- | --- | --- | --- | --- | --- |
|  |  | Window  count | Non-overlapped length (bp) | Window  count | Non-overlapped length (bp) | Percentage |
| Felidae | 2,419,212,910 | 236,332 | 2,404,232,357 | 112,821 | 1,128,179,303 | 46.92 % |
| Hominidae | 3,088,269,832 | 267,977 | 2,732,432,232 | 93,165 | 931,656,495 | 34.10 % |
| Bovidae | 2,660,906,405 | 257,230 | 2,616,313,800 | 87,923 | 879,223,575 | 33.61 % |

**Table S44. GO enrichment of shared genes in the highly conserved regions of Felidae, Hominidae, and Bovidae.** Only GO categories with *P* < 1.00E-08 are shown.

| Term | Count | *P*-value | FDR |
| --- | --- | --- | --- |
| GO:0031981~nuclear lumen | 476 | 8.08E-31 | 1.23E-27 |
| GO:0070013~intracellular organelle lumen | 557 | 2.53E-30 | 3.87E-27 |
| GO:0031974~membrane-enclosed lumen | 576 | 2.54E-30 | 3.88E-27 |
| GO:0043233~organelle lumen | 564 | 1.95E-29 | 2.99E-26 |
| GO:0005654~nucleoplasm | 307 | 5.02E-24 | 7.67E-21 |
| GO:0030528~transcription regulator activity | 470 | 1.84E-17 | 3.11E-14 |
| GO:0045449~regulation of transcription | 751 | 5.13E-16 | 1.05E-12 |
| GO:0044451~nucleoplasm part | 195 | 1.20E-15 | 1.87E-12 |
| GO:0006350~transcription | 618 | 1.12E-14 | 2.12E-11 |
| GO:0043232~intracellular non-membrane-bounded organelle | 682 | 1.37E-14 | 2.09E-11 |
| GO:0043228~non-membrane-bounded organelle | 682 | 1.37E-14 | 2.09E-11 |
| GO:0005730~nucleolus | 229 | 1.91E-14 | 2.92E-11 |
| GO:0051603~proteolysis involved in cellular protein catabolic process | 215 | 1.56E-13 | 2.96E-10 |
| GO:0006357~regulation of transcription from RNA polymerase II promoter | 250 | 2.65E-13 | 5.02E-10 |
| GO:0044257~cellular protein catabolic process | 215 | 2.77E-13 | 5.24E-10 |
| GO:0043632~modification-dependent macromolecule catabolic process | 206 | 4.50E-13 | 8.52E-10 |
| GO:0019941~modification-dependent protein catabolic process | 206 | 4.50E-13 | 8.52E-10 |
| GO:0030163~protein catabolic process | 218 | 1.32E-12 | 2.50E-09 |
| GO:0045941~positive regulation of transcription | 201 | 1.89E-12 | 3.58E-09 |
| GO:0045893~positive regulation of transcription, DNA-dependent | 175 | 3.75E-12 | 7.10E-09 |
| GO:0016568~chromatin modification | 114 | 4.37E-12 | 8.27E-09 |
| GO:0051254~positive regulation of RNA metabolic process | 175 | 8.43E-12 | 1.60E-08 |
| GO:0051276~chromosome organization | 176 | 9.51E-12 | 1.80E-08 |
| GO:0010628~positive regulation of gene expression | 203 | 1.18E-11 | 2.23E-08 |
| GO:0010604~positive regulation of macromolecule metabolic process | 279 | 1.23E-11 | 2.33E-08 |
| GO:0044265~cellular macromolecule catabolic process | 242 | 2.17E-11 | 4.10E-08 |
| GO:0003677~DNA binding | 643 | 3.42E-11 | 5.77E-08 |
| GO:0045935~positive regulation of nucleobase, nucleoside, nucleotide and  nucleic acid metabolic process | 213 | 4.14E-11 | 7.84E-08 |
| GO:0003700~transcription factor activity | 303 | 4.91E-11 | 8.29E-08 |
| GO:0010557~positive regulation of macromolecule biosynthetic process | 221 | 4.98E-11 | 9.43E-08 |
| GO:0007049~cell cycle | 254 | 6.33E-11 | 1.20E-07 |
| GO:0051173~positive regulation of nitrogen compound metabolic process | 217 | 1.02E-10 | 1.94E-07 |
| GO:0022402~cell cycle process | 192 | 6.33E-10 | 1.20E-06 |
| GO:0009891~positive regulation of biosynthetic process | 227 | 9.19E-10 | 1.74E-06 |
| GO:0031328~positive regulation of cellular biosynthetic process | 224 | 1.07E-09 | 2.03E-06 |
| GO:0045944~positive regulation of transcription from RNA polymerase II  promoter | 135 | 2.47E-09 | 4.68E-06 |
| GO:0009057~macromolecule catabolic process | 248 | 2.78E-09 | 5.26E-06 |
| GO:0006325~chromatin organization | 136 | 4.91E-09 | 9.30E-06 |
| GO:0005829~cytosol | 359 | 5.49E-09 | 8.39E-06 |

**Table S45. KEGG pathway enrichment of shared genes in the highly conserved regions of Felidae, Hominidae, and Bovidae**

| Term | Count | *P*-value | FDR |
| --- | --- | --- | --- |
| hsa04110:Cell cycle | 45 | 1.94E-04 | 0.24 |
| hsa05200:Pathways in cancer | 97 | 2.52E-04 | 0.31 |
| hsa05211:Renal cell carcinoma | 28 | 6.68E-04 | 0.83 |
| hsa03050:Proteasome | 21 | 7.75E-04 | 0.96 |
| hsa04340:Hedgehog signaling pathway | 23 | 0.0016 | 1.92 |
| hsa04120:Ubiquitin mediated proteolysis | 45 | 0.0018 | 2.18 |
| hsa03018:RNA degradation | 23 | 0.0020 | 2.50 |
| hsa04914:Progesterone-mediated oocyte maturation | 30 | 0.0047 | 5.74 |
| hsa04114:Oocyte meiosis | 36 | 0.0059 | 7.12 |
| hsa00230:Purine metabolism | 47 | 0.0059 | 7.13 |

**Table S46. GO enrichment of Felidae-specific genes in the highly conserved regions**

| Term | Count | *P*-value | FDR |
| --- | --- | --- | --- |
| GO:0006811~ion transport | 84 | 4.56E-06 | 0.008 |
| GO:0005261~cation channel activity | 40 | 1.10E-05 | 0.018 |
| GO:0046873~metal ion transmembrane transporter activity | 45 | 1.30E-05 | 0.021 |
| GO:0016892~endoribonuclease activity, producing 3'-phosphomonoesters | 9 | 1.79E-05 | 0.029 |
| GO:0016894~endonuclease activity, active with either ribo- or deoxyribonucleic  acids and producing 3'-phosphomonoesters | 10 | 2.39E-05 | 0.038 |
| GO:0005216~ion channel activity | 49 | 4.01E-05 | 0.064 |
| GO:0005509~calcium ion binding | 95 | 4.36E-05 | 0.069 |
| GO:0004522~pancreatic ribonuclease activity | 8 | 4.56E-05 | 0.073 |
| GO:0004521~endoribonuclease activity | 13 | 5.51E-05 | 0.088 |
| GO:0022836~gated channel activity | 41 | 7.70E-05 | 0.12 |
| GO:0022838~substrate specific channel activity | 49 | 8.64E-05 | 0.14 |
| GO:0006812~cation transport | 61 | 9.06E-05 | 0.16 |
| GO:0015267~channel activity | 50 | 1.04E-04 | 0.16 |
| GO:0022803~passive transmembrane transporter activity | 50 | 1.11E-04 | 0.18 |
| GO:0034702~ion channel complex | 30 | 1.23E-04 | 0.18 |
| GO:0030001~metal ion transport | 53 | 1.25E-04 | 0.22 |
| GO:0044459~plasma membrane part | 190 | 1.78E-04 | 0.26 |
| GO:0034703~cation channel complex | 22 | 2.03E-04 | 0.29 |
| GO:0031226~intrinsic to plasma membrane | 114 | 2.40E-04 | 0.35 |
| GO:0005887~integral to plasma membrane | 111 | 3.46E-04 | 0.50 |
| GO:0004519~endonuclease activity | 18 | 4.14E-04 | 0.66 |
| GO:0045177~apical part of cell | 26 | 4.28E-04 | 0.61 |
| GO:0004540~ribonuclease activity | 14 | 4.56E-04 | 0.72 |
| GO:0016051~carbohydrate biosynthetic process | 18 | 6.13E-04 | 1.10 |
| GO:0015672~monovalent inorganic cation transport | 37 | 0.0010 | 1.87 |
| GO:0005886~plasma membrane | 297 | 0.0012 | 1.74 |
| GO:0050877~neurological system process | 107 | 0.0013 | 2.39 |
| GO:0016324~apical plasma membrane | 20 | 0.0015 | 2.19 |
| GO:0034637~cellular carbohydrate biosynthetic process | 13 | 0.0017 | 3.02 |
| GO:0007601~visual perception | 27 | 0.0022 | 3.80 |
| GO:0050953~sensory perception of light stimulus | 27 | 0.0022 | 3.80 |
| GO:0034706~sodium channel complex | 6 | 0.0024 | 3.37 |
| GO:0022843~voltage-gated cation channel activity | 21 | 0.0024 | 3.82 |
| GO:0004518~nuclease activity | 22 | 0.0026 | 4.02 |
| GO:0031224~intrinsic to membrane | 412 | 0.0026 | 3.65 |
| GO:0007267~cell-cell signaling | 58 | 0.0033 | 5.81 |
| GO:0031420~alkali metal ion binding | 28 | 0.0037 | 5.78 |
| GO:0055085~transmembrane transport | 55 | 0.0043 | 7.45 |
| GO:0007268~synaptic transmission | 33 | 0.0044 | 7.71 |
| GO:0019226~transmission of nerve impulse | 37 | 0.0054 | 9.24 |
| GO:0006816~calcium ion transport | 19 | 0.0057 | 9.80 |

**Table S47. KEGG pathway enrichment of Felidae-specific genes in the highly conserved regions**

| Term | Count | *P*-value | FDR |
| --- | --- | --- | --- |
| hsa04360:Axon guidance | 20 | 0.0054 | 6.42 |

**Table S48. GO enrichment of Hominidae-specific genes in the highly conserved regions**

| Term | Count | *P*-value | FDR |
| --- | --- | --- | --- |
| GO:0043235~receptor complex | 26 | 5.65E-04 | 0.83 |
| GO:0044429~mitochondrial part | 91 | 6.04E-04 | 0.89 |
| GO:0034364~high-density lipoprotein particle | 10 | 8.15E-04 | 1.20 |
| GO:0055085~transmembrane transport | 89 | 9.16E-04 | 1.68 |
| GO:0005887~integral to plasma membrane | 160 | 0.0023 | 3.33 |
| GO:0005886~plasma membrane | 456 | 0.0026 | 3.73 |
| GO:0033700~phospholipid efflux | 6 | 0.0027 | 4.87 |
| GO:0031090~organelle membrane | 148 | 0.0030 | 4.39 |
| GO:0005789~endoplasmic reticulum membrane | 45 | 0.0035 | 5.00 |
| GO:0016125~sterol metabolic process | 22 | 0.0035 | 6.33 |
| GO:0034361~very-low-density lipoprotein particle | 8 | 0.0037 | 5.42 |
| GO:0034385~triglyceride-rich lipoprotein particle | 8 | 0.0037 | 5.42 |
| GO:0044432~endoplasmic reticulum part | 55 | 0.0039 | 5.58 |
| GO:0007155~cell adhesion | 102 | 0.0039 | 7.05 |
| GO:0022610~biological adhesion | 102 | 0.0041 | 7.33 |
| GO:0031226~intrinsic to plasma membrane | 161 | 0.0041 | 5.96 |
| GO:0001570~vasculogenesis | 12 | 0.0044 | 7.74 |
| GO:0001819~positive regulation of cytokine production | 20 | 0.0045 | 8.01 |
| GO:0004713~protein tyrosine kinase activity | 31 | 0.0048 | 7.57 |
| GO:0008092~cytoskeletal protein binding | 76 | 0.0049 | 7.65 |
| GO:0005739~mitochondrion | 145 | 0.0052 | 7.45 |
| GO:0005516~calmodulin binding | 27 | 0.0058 | 9.01 |
| GO:0005740~mitochondrial envelope | 63 | 0.0066 | 9.35 |

**Table S49. GO enrichment of Bovidae-specific genes in the highly conserved regions**

| Term | Count | *P*-value | FDR |
| --- | --- | --- | --- |
| GO:0007608~sensory perception of smell | 82 | 2.44E-16 | 4.00E-13 |
| GO:0007606~sensory perception of chemical stimulus | 87 | 3.36E-16 | 6.00E-13 |
| GO:0004984~olfactory receptor activity | 81 | 1.36E-15 | 2.11E-12 |
| GO:0007166~cell surface receptor linked signal transduction | 210 | 5.90E-13 | 1.06E-09 |
| GO:0007186~G-protein coupled receptor protein signaling pathway | 143 | 1.78E-12 | 3.19E-09 |
| GO:0007600~sensory perception | 111 | 1.02E-11 | 1.83E-08 |
| GO:0050890~cognition | 113 | 2.54E-09 | 4.57E-06 |
| GO:0050877~neurological system process | 137 | 1.70E-08 | 3.06E-05 |
| GO:0005886~plasma membrane | 306 | 1.55E-04 | 0.22 |
| GO:0044427~chromosomal part | 44 | 9.19E-04 | 1.30 |
| GO:0030141~secretory granule | 25 | 0.0011 | 1.63 |
| GO:0000785~chromatin | 26 | 0.0023 | 3.21 |
| GO:0043120~tumor necrosis factor binding | 5 | 0.0025 | 3.82 |
| GO:0000786~nucleosome | 12 | 0.0032 | 4.44 |
| GO:0005694~chromosome | 48 | 0.0032 | 4.53 |
| GO:0004499~flavin-containing monooxygenase activity | 4 | 0.0033 | 5.07 |
| GO:0031091~platelet alpha granule | 11 | 0.0041 | 5.66 |
| GO:0005576~extracellular region | 166 | 0.0043 | 5.89 |
| GO:0019932~second-messenger-mediated signaling | 29 | 0.0045 | 7.74 |
| GO:0016165~lipoxygenase activity | 4 | 0.0062 | 9.39 |

**Table S50. KEGG pathway enrichment of Bovidae-specific genes in the highly conserved regions**

| Term | Count | *P*-value | FDR |
| --- | --- | --- | --- |
| hsa04740:Olfactory transduction | 83 | 1.20E-17 | 1.47E-14 |
